# Abstracts of papers presented at the 1993 Pittsburgh Conference

**DOI:** 10.1155/S1463924693000148

**Published:** 1993


Journal of Automatic Chemistry, Vol. 15, No. 3 (May-June 1993), pp. 85-120

### Abstracts of papers presented at the 1993 Pittsburgh Conference

### Cluster analysis of secondary ion mass spectra

*Peng Zheng and Peter de B. Harrington, Ohio University, Center for Intelligent Chemical Instrumentation, Department of Chemistry, Clippinger Laboratories, Athens, OH 45701-2979*

High resolution time-of-flight secondary ion mass spectrometry (HR TOF-SIMS) is a powerful analytical technique. Features of this technique include high resolution, surface sensitivity and low primary ion dose, which prevent beam damage to the sample and make HR TOF-SIMS suitable for organic analysis. The high mass resolution and complicated fragmentation patterns yield intricate spectra which may be difficult to interpret visually. Chemometric methods, such as cluster analysis and principal component analysis, may be used to characterize samples and to classify unknown materials.

The prerequisite for many multivariate data analytical techniques requires alignment of the data variables. If uncertainties exist, or the precision of the variable measurement (or the resolution in spectrum) is unknown, this task may become difficult. Once the variables have been aligned, the data may be represented in a matrix format in which the rows correspond to spectra and the columns correspond to mass measurements.

Clustering methods are numerical procedures for grouping similar objects. Principal component analysis is a matrix technique that can be applied to compress data sets and to extract multivariate information. A useful algorithm has been developed for clustering high resolution time-of-flight secondary ion mass spectra of steel samples based on mass resolution. Principal component analysis is applied as a useful display method.

### Chromatography data interpretation using three-dimensional, multivariate visualization

*F. O. Geiser, Geiser Scientific, Inc., 287-A Wilson Ave., Glen Mills, PA 19342, R. D. Ricker, Rockland Technologies, Inc., 538 First State Blvd., Newport, DE 19804, C. Golt, Agriculture Experiment Station, University of Delaware, Newark, DE 19716, J. D. Justice, Justice Innovations, Inc., 465 El Capitan Place, Palo Alto, CA 94306 and J. W. Duffy, Echo Data, Inc., 1010 N. State St., Orem, UT 84057*

The objective was to verify whether principal-component graphical analysis, as automated in Justice Innovations' *Chrom Perfect* and Echo Data's *DataMax* PC-based software, could accurately represent the complex relationships in *n*-dimensional chromatography data. Examples are shown in which as many as 35 highly-correlated variables were simultaneously visualized in a single 3-D map using cosines of the correlation coefficients as vector angles. Applications include the validation of system-suitability parameters and the identification of ionic strength and pH effects in size exclusion chromatography.

### A critical evaluation of computer assisted pattern recognition in arson analysis

*P. Koussiafes and W. Bertsch, Department of Chemistry, The University of Alabama, Box 870336, Tuscaloosa, AL 35487-0336; G. Holzer, School of Biology, Georgia Institute of Technology, Atlanta, GA 30332*

The term 'arson analysis' is a misnomer that describes analytical methodology concerned with the determination of accelerants in fire debris. Several techniques for the isolation of volatile organics from debris have been established. Arson analysis is generally based on GCs and, lately, also on GC/MS. In competent hands, goods results in the form of chromatograms can readily be established but data interpretation remains a serious challenge in establishing an accelerant.

ASTM method E 1387-90 describes standard test methods for combustibles in fire debris. It states minimum requirements for class identification. In practice, it is rather difficult to identify particular groupings of peaks in the presence of large amounts of interferences. Comparison of chromatographic profiles between the sample and a series of standards is also difficult because of external factors such as loss due to evaporation in the heat of a fire. The term 'pattern recognition' is frequently used in this context, even though the minimum statistical requirements are seldom met.

The authors have exploited the selectivity of GC/MS, a situation which, unfortunately, is not often encountered in the average laboratory. To be more in line with the majority of laboratories, several software packages that operate on FID generated databases have recently been tested. The systems work very well for conventional chromatograms where the degree of interference is manageable. Some surprising and unexpected failures also materialized. This presentation pointed out the factors that are important in computer assisted evaluations; several examples were presented.

### Factor analysis of fiber-optic Raman spectroscopy data from thermoset polymers during the curing process

*Melinda K. Higgins, Jeffrey F. Aust, Stephen L. Morgan and Michael L. Myrick, Department of Chemistry & Biochemistry, University of South Carolina, Columbia, SC 29208*

Raman spectroscopy using fibre-optic probes has been employed to monitor the curing process for industrial bonding agents. The fibre-optic probe is sandwiched between glass fibre composites preimpregnated with polyimide bonding agents. Raman spectra obtained by monitoring the curing process represent mixtures of several chemical components. Spectral subtraction or least squares fitting of reference spectra is not adequate to resolve mixture spectra for such a real-time system. Unpredictable chemical changes in polymeric structure complicate spectral interpretation. Additionally, reference spectra of pure components are not usually available.

Work with factor analysis of fibre-optic Raman data at the authors' department has focused on obtaining information on the varying compositions of chemical components during the curing process. Significant changes occurring in Raman spectra at 30 min intervals are not simple increases or decreases in the magnitude of single peaks, but rather a combination of many changes that mirror the complex chemical reactions taking place during curing. Principal component analysis (PCA) of this data reduces the dimensionality of the spectral data to just a few principal components which retain the variability related to chemical changes. A PCA plot of these Raman spectra may help to elucidate the nature of these chemical changes during the curing process. A principal component regression (PCR) model of these changes can also be used to monitor the percentage cure of bonding agent.

### The importance of ultra-pure water used in the analytical laboratory

*Jim Krol, Kitty K. Siu, Cindy S. Long and Tom Wheat, Millipore Corporation, 80 Ashby Road, Bedford, MA*

Ultra-pure dionized water is used in the laboratory for the preparation of mobile phases and eluents for analytical instruments such as HPLC, IC, CE, AA and ICP, and is used as a solvent for sample preparation, quantitative standards and blanks. With the advancement of analytical techniques, an increasing number of instruments have the capability to measure organics and inorganic ions in samples at ultra-low µg/l (ppb) levels. The quality of the ultra-pure water thus plays an important role in the ability to consistently and reproducibility achieve these low detection limits.

Single-step water purification systems, such as distillation or conventional deionization, are not adequate water sources for today's high technology instruments utilizing reverse phase gradients with detection at less than 220 nm, ion chromatography with conductivity detection, and inorganic capillary electrophoresis. Organic and ionic impurities present in water will cause long term 'column fouling' which in turn yields unstable baselines, decreased column capacity lowering 'k', and 'ghost peaks' which may obscure key components in the sample.

The analytical techniques used to analyse the quality of ultra-pure water, HPLC, IC, CIA, microbiology and Total Organic Carbon (TOC) were discussed. These techniques were used to evaluate the quality of water produced by deionization systems and a distillation system, from feed water to point-of-use, and HPLC bottled water. The measured quality of each of these types of water was related to analytical performance in each of the techniques.

### The importance of workflow analysis in LIMS selection

*Terry V. Iorns, National Pharmaceutical Industry Practice, Coopers & Lybrand, One Sylvan Way, Parsippany, NJ 07054,*

A LIMS or other laboratory automation system is a tool to help providers and users of information improve their effectiveness. Benefits of a LIMS implementation usually include increasing productivity, improving data and information quality, shortening product development cycles, meeting regulatory requirements, and improving management capabilities.

Workflow analysis during later stages of project is used to squeeze a few more benefits out of an ongoing process, hoping to extend the life of existing systems. Often, existing system constraints make it difficult to achieve the needed improvements.

Workflow analysis is a more valuable technique during system planning and selection phases. For a successful project, it is important to both optimize the process and automate the activities. During the early phases of an automation project, it is possible to be more flexible in identifying new ways of carrying out laboratory activities. After a system is selected and implemented, constraints may exist that make significant workflow improvements more difficult.

Experiences from recent projects were highlighted. Examples include:

- Implementing continuous analysers in a batch-oriented environment.
- Eliminating method steps in a regulated environment.
- Moving toward a paperless laboratory.
- Changing the way work assignments are made.
- Streamlining data checking and approval steps.

### Contracting for a LIMS: practical advice for reaching your laboratory information management system goals

*Randall R. Stein, Chesapeake Software, Inc., Brandywine Five Building, Routes 1 and 202, Chadds Ford, PA 19317*

The purchase of information systems has not been a traditional laboratory activity, so many laboratory organizations are entering new territory when contracting for a LIMS. This paper discussed practical techniques for protecting organizations against excessive expenditures for a system that does not meet the organization's needs.

Areas discussed included:

(1) Specification:
Getting the features wanted.
Integrating with existing and planned systems.

The important trade-off of company standards.
Expressing requirements without being a software expert.

(2) Selection:
Making sure the vendor is capable of doing the job.
Categories of vendor capabilities.

(3) Pricing:
Avoiding high-cost 'out of scope' items.
Comparing vendor costs.
Important terms and conditions.
Avoiding creeping hardware obsolescence.

(4) Assurance:
Making sure the system will remain useful for long enough to get a payback.
Getting money back if the system is not what was wanted.
Handling software defects.

The material discussed was upon the author's experience as a software vendor.

### Implementation of LIMS network at Atec Associates, Inc.

*Arun K. Bhattacharya, Corporate Director, Analytical Services, ATEC Associates, Inc., 8665 Bash Street, Indianapolis, IN 46256 and Brude Widener, ATEC Associates, Inc., 5150 E. 65th Street, Indianapolis, IN 46220*

ATEC Associates, Inc. was established in 1958 and provides environmental, geotechnical, and materials testing, consulting, and engineering services. The Analytical Testing Division includes five chemistry laboratories located at Indianapolis, Indiana; Atlanta, Georgia; Dallas, Texas; Columbia, Maryland; and Highland, Indiana, and a mixed waste laboratory at Oak Ridge, Tennessee. ATEC is also the sole Environmental Analytical Laboratory contractor for General Motors' technical facility at Warren, Michigan.

ATEC's LIMS implementation started in 1990 by acquiring the SAM Software from Radian Corporation. The hardware includes a PC based Novell Network. The original software has undergone several phases of modifications to arrive at the current customized system. In this paper some of the details of the debugging and improvement with feedback from clients, laboratory users, and management staff is described.

Currently, ATEC's Indianapolis laboratory is using the LIMS to produce all their organic and inorganic chemistry reports. Steps have now been taken to install our second LIMS at General Motors' Technical Center facility located in Warren, Michigan. 9600 BAUD Rate Modem Telephone Systems are employed to initiate the creation of ATEC's LIMS National Network. The current LIMS package allows special tests to be created. Services to provide data in customized format to meet any unique requirements are also offered.

### Financial impact of GALP compliance on sampled environmental, chemical, and pharmaceutical laboratories

*Sandy Weinberg, CVP, Janie S. Spelton, CVP and Philip E. Sax, CVP, Weinberg Research Institute, 1440 Conchester Hwy., Boothwyn, PA 19061*

The newly released Environmental Protection Agency's 'Good Automated Laboratory Practices' (GALPs) identify new organizational, testing, documentation, and archive recommendations for contract and independent laboratories submitting reports to a variety of Federal agencies. To determine the financial impact of compliance, the researchers visited a sample of seven laboratories of differing sizes, geographical locations, and purposes. In a two-week in-depth study of each laboratory, an assessment of the long and short term costs of modification in physical plant, equipment, personnel, and procedure led to a composite estimate of the cost of GALP compliance. This paper identified the kinds of operational and functional modifications that the GALPs imply; estimated the cost of those modifications based upon actual field observation; and defended the potentially surprising conclusion that, when factored over a five year period, the end result of GALP compliance is a small saving in operating and investment costs, rather than any increase at all.

### Development of a prediction scheme for raw material identification and quality assessment using near IR spectroscopy

*Julia A. Giambra, Department of Chemistry, State University of New York, College at Fredonia, NY 14063 and E. W. Ciurczak, Department of Chemistry, Seton Hall University, S. Orange, NJ 07079*

A combination of Spectral Matching and Principal Components algorithms were used for the identification and quality assessment of incoming raw materials at a major pharmaceutical company. This world-wide study was performed with the participation of 16 subsidiaries. In addition to a description of the commercially available software used, the criteria for choosing samples and wavelength ranges were discussed. Criteria for determining what constitutes a 'good' material and how these criteria are related in various countries was also discussed.

Incoming raw materials are currently tested using a statistical method which, by chances, may allow an incorrect sample to get through. This method is both antiquated and time consuming. Using raw material learning sets with the near IR, 100% testing may be done with less time and energy.

### A new approach for quantifying simethicone

*Denise E. Grzybowski and Stephen L. Monfre, NIR Systems, Inc., 12101 Tech Road, Silver Spring, MD 20904*

Simethicone is one of the active ingredients in antacid/anti-gas materials. As with any active ingredient found in drugs, it is important to monitor the concentration of active ingredient. Current compendial methods approved by the FDA and USP involve the extraction using carbon tetrachloride, a known carcinogen. Furthermore, recent legislation has limited the use of carbon tetrachloride in extraction analyses, thereby rendering the compendial method ineffective.

A new approach for quantifying the concentration of simethicone has been developed using near-infrared (NIR) spectrophotometry. The primary NIR analysis method has been developed based on liquid emulsion materials, where the concentration of the simethicone is typically around 4 mg/gram. The accuracy is similar to that which is obtained from the primary analysis method, while the precision appears to be better.

The paper described the way samples are prepared and analysed. The sampling techniques and sampling errors which were encountered were discussed in detail. Preliminary results from the analysis of antacid/anti-gas tablets was also presented.

### Monitoring fermentation broths with a fibre-optic near-IR sensor: a preliminary study

*Chris W. Brown, Yue Li and Jie Lin, Department of Chemistry, University of Rhode Island, Kingston, RI 02881*

Potentially, fibre optic sensors are ideal for following the status of chemical reactions. Light can be transferred between a spectrometer and a probe or sensor in a reaction vessel over optical fibres; the instrument and the reaction vessel can be separated by 25 meters or more. Moreover, multiplexing techniques make it possible to monitor several reacting vessels simultaneously.

As a preliminary study for using fibre optic sensors to follow fermentation reactions, spectra have been measured and principal component regression performed on both methanol and ethanol solutions in $CCl_4$ over a range from 0.1 to 100% (V/V). The main problem of using near-IR to quantitatively monitor most fermentation broths is that the reactants and products are susceptible to hydrogen bonding which can alter the spectra to produce non-linear dependence with concentration. Thus, in this preliminary study, several simple models were investigated for handling the non-linear dependencies.

A simple model system of xylose fermentation to ethanol and xylitol was also investigated. Spectra of all possible components over all possible concentration ranges were measured. Initially, we measured spectra of the pure components in inert solvents. This was followed by a multicomponent investigation and finally by the analysis of an actual broth with a fibre optic interface.

### Multielement trace analysis of discrete microsamples of solutions by glow discharge emission spectrometry with a photodiode array

*C. A. Morgan, C. Davis and J. D. Winefordner, University of Florida, Gainesville, FL 32611*

Analytical techniques for multielement trace analysis of solutions typically involve continuous sample introduction and therefore require large sample volumes. There are, however, many situations where only microsamples may be available for analysis, as in the archeological, forensic and the biomedical fields. The need for an analytical technique capable of trace and ultratrace multielement analysis of discrete nanolitre samples is clear.

Over the years, the hollow cathode glow discharge has been proven to be an efficient atom source for ultratrace analysis of solution residues. Coupling the hollow cathode glow discharge with a photodiode array extends the technique from single element to multielement capability.

In the authors' laboratory, the use of a microcavity hollow cathode to boost the emission signal is under study. Several multielement schemes are being investigated and parametric evaluations are being conducted to determine optimum compromise conditions. Results of evaluations of current, voltage, pressure, flow rate, cathodic material and hollow cathode geometry were presented. In addition to this, analytical figures of merit such as detection limits, precision, S/B and S/N were discussed.

### Novel approaches to high pressure microwave sample preparation: focused bomb vessel and on-line digestion

*Henryk Matusiewicz, Politechnika Poznańska, Department of Analytical Chemistry, 60-965 Poznań, Poland*

The complete digestion of sample is now considered to be the proper approach required to achieve reproducible and accurate results using elemental instrumental analytical methods. Present state-of-the-art wet digestion utilizes closed decomposition and dissolution systems coupled with microwave radiation to assist in digestion.

This lecture reflected current interest in focused high pressure microwave digestion approaches that exceed the operational capabilities of existing microwave oven digestion arrangements. An integrated system offers very efficient energy transfer from the microwave generator to the sample and acid(s) and provides both continuous (unpulsed) low-power, as well as high-power microwave energy.

In spite of the advantages offered by closed vessel microwave digestions, sample preparation still remains a multi-step and labour-intensive procedure. At present, particular attention is being paid to the possibilities of on-line continuous and stopped-flow microwave sample pretreatment. The feasibility of on-line, high pressure sample digestion in a microwave cavity instead of microwave oven was discussed.

### Application of microwave heating to traditional sample preparation techniques: saponification and solvent extraction

*L. B. Jassie, CEM Corporation, Matthews, NC 28106; S. A. Margolis, N. E. Craft and M. J. Hays, Organic Analytical Research Division, Chemical Science and Technology Laboratory, National Institute of Standards and Technology, Gaithersburg, MD 20899*

In analytical chemistry we are familiar with microwave energy assisted acid dissolution for the preparation of inorganic and organic materials for the analysis of their elemental content. Since the introduction in 1985 of microwave dissolution systems featuring *in situ* temperature measurement and external pressure sensing, microwave equipment has steadily evolved. The newest microwave instruments are true systems because both temperature and pressure feedback control of the energy supply are now an integral part of the unit. Control of temperature sensitive reactions and sample preparations are now easier and more reliable with this new technology. This presentation discussed research efforts in two classical sample preparation procedures that require carefully thermostated conditions: alkaline hydrolysis of foods, and the organic solvent extraction of sediments.

Vitamins A and E were recovered from fortified food matrices, such as infant formula, after microwave saponification for 10 min at 45°C in alcoholic potassium hydroxide. The addition of pyrogallol as an antioxidant was necessary to inhibit oxidation of tocopherols under alkaline conditions. Hexane extracts containing the vitamins were dried under nitrogen and redissolved in ethanol for analysis by liquid chromatography (LC). Both vitamins were quantitatively measured using reversed-phase LC by isocratic methanol elution from a C-18 column. Vitamin A was also recovered quantitatively under more aggressive microwave heating conditions of as little as 2 min; unfortunately, vitamin E was degraded under these more vigorous conditions.

Closed vessel microwave extraction of polycyclic aromatic hydrocarbons (PAHs) from sediments has been examined as an alternative to Soxhlet extraction using an approach similar to that used for the extraction of stabilizers from plastics and polymers. In applying this technique to sediments, methylene chloride was used as the extractant to determine PAHs. With the Soxhlet method, the extraction temperature is fixed at ~40°C. Closed vessel microwave extractions for 15 min at both 40°C and 100°C, as well as, a 4 h extraction at 40°C gave results that are comparable to traditional 16 h methylene chloride Soxhlet extractions. To avoid the use of halogenated solvents for the measurement of PAHs in sediments, alternate extractants also were examined using both Soxhlet and microwave techniques.

### Microwave sample preparation – the state-of-the-art

*Stephen J. Haswell, School of Chemistry, University of Hull, Hull HU6 7RX, UK*

Microwave sample preparation, over the last ten years, has become a popular and attractive method sample preparation particularly in the field of inorganic analytical chemistry. The technique has almost exclusively focused on batch methodology with wet oxidations and sample hydrolysis being carried out in either pressurized or unpressurized systems. A considerable literature base has evolved around such techniques describing the fundamental and practical aspects of the methodology. Work over the past five years in the author's laboratory has taken a radical look at microwave assisted reactions and a summary of these findings of this work and that of contemporary research was presented. The main theme of this work has been to modify the traditional batch digestion methods to incorporate the benefits of Flow Injection Analysis (FIA) methodology. In addition to describing the practicalities of performing such novel procedures, the presentation focused also on the underlying mechanisms which are unique to microwave enhanced reactions in such geometries.

To date the main application of the technique has been directed towards on-line sample digestion of inorganic and organic matrices and results will be presented for both atomic spectroscopic and colorimetric coupled detection for a range of trace elements including P and N. More recently the technique has been used to carry out on-line chemical derivatization producing for example methyl esters of fatty acids for direct coupling to GC instrumentation. Clearly the opportunities to perform controlled on-line synthetic reactions affords exciting possibilities for future analytical methodology.

### Control of autosampler-equipped gas chromatographs using personal computers

*William W. Gardiner, Research and Development, Analytech, A Division of Laboratory Consulting Sources, Inc., 1197 Spur 138, Jonesboro, GA 30236*

The use of PC operating system batch files in conjunction with spreadsheet software to output control data useful for controlling autosampler-equipped gas chromatographs was demonstrated. MS-DOS batch files when used in conjunction with the native capabilities of Lotus 1-2-3 release 2.x-compatible software and proprietary chromatography software sub-routines provide sufficient programming capability to (1) automatically identify and quantify target analytes on a pre-calibrated GC system; (2) determine the need and value, if any, of subsequent dilutions as may be required to meet contract-required detection limits (CRDLs) on the low end of the linear concentration range of the analytical system and not to exceed system mass capacity on the high end.

Operation of the software is demonstrated across several operating system platforms, including MS-DOS release 5.0, Windows 3.1, OS/2, and as a stand-alone (Baled) application. The capability is currently in laboratory operation for the organic priority pollutant volatiles, phenols, base/neutrals, and pesticides, for all standard and wide-bore capillary columns (0.25 mm, 0.35 mm, and 0.53 mm column ID's), and for FID, ElCD, and PID detectors. This capability expands upon the analyte identification, verification, and quantification (AIVQ) software developed in our laboratory and previously described. Data is collected via a 16-bit analog/digital interface, then integrated using Labtech Chrom on a second, 386-class computer. The quantitative results were tabulated following optional opportunities to the authorized user to review the system parameters, detector mass limits, and column mass capacities.

Dilution information is exported via ASCII text or numbers files which may be ported to an appropriately configured autosampler in order either to trigger selection of a 'best guess' dilution from a set of previously dilutions or to trigger preparation of such a dilution using autosampler equipment with that capability. Sample macros were presented which demonstrated both fully automated operation based on default values and optimization options pre-selected by the user and user-interruptible and modifiable operation.

### Chromatographic data acquisition advances in the UNIX networked environment

*Derek F. Rhodes, Peter Yendle, Kevin Smith, Steven Kemp and Richard Lane, VG Data Systems, Division of Fisons Instruments, 32 Commerce Center, Danvers, MA 01923*

As the UNIX operating system becomes more widespread in a variety of computer applications, the need for a chromatographic software application in this environment has become paramount within many analytical laboratories. VG Data Systems, a division of Fisons Instruments, has been actively investigating data acquisition for chromatographic instrumentation utilizing the UNIX operating system. As a result, VG Data Systems has successfully implemented data acquisition devices on a networked platform communicating to UNIX clustered workstations. The software application on these workstations utilizes the standard MOTIF X-Windowing interface to allow viewing of any and/or all chromatographic activity on the network. This environment allows real time manipulation of chromatographic data for peak integration, method development and instantaneous result calculation. Further, chromatographic activity can be monitored utilizing Microsoft Windows connected to the network via conventional DOS computers by employing appropriate X-Terminal emulation.

The advantages of utilizing a GUI (Graphical User Interface) have been well documented, in the ease of use and reduction of the learning curve for subsequent products under the same user interface. Therefore, the software applied to this networked environment adheres to the Open Software Foundation guidelines for a Windows Graphical User Interface.

The development, application and implementation of this powerful software in a networked client/server environment was presented. The development of this software requires the use of an advanced code management system, along with strict adherence to regulatory guidelines and standards. This was discussed in relation to the application of the product in present day regulated and non-regulated laboratories. Concerns of security, validation, file protection, audit trail and data ownership, were addressed.

The arena of networked data acquisition research is a very active one, with constant improvements and more efficient protocols being introduced. The progression of new networking protocols, as well as implementation of current industry standards, was discussed in relation to the changing implementation of such products.

### A multi-user, multi-hardware, networked chromatography workstation on the Macintosh

*David Willcox, WillStein Software, Inc., 1723 Elmwood Avenue, Wilmette, IL 60091*

The chief advantages of the software presented include: support for a variety of GCs and LCs, use of a non-dedicated Macintosh to collect data over a network, and automatic communication of data to other software programs.

First, supporting several GCs and LCs allows a system to be designed for a particular set of needs. For example, some users may wish to use a single low cost A/D converter for several chromatographs. Others can use the RS ≅ 232 option for GCs like the HP 5890. No single A/D converter can meet all users needs of accuracy, speed, flexibility, and price.

Second, off the shelf networking devices can be used to connect any GC/LC to a LocalTalk or EtherNet network. Data can then be collected in the background from an office Macintosh even though the chromatograph may be located in a distant laboratory, even while the Macintosh is used for other purposes.

Finally, data may be communicated to ofther software programs for inclusion in reports or statistical analyses, or the data can be used for online process control. A variety of methods are supported for this. The most exciting is the use of Apple Events which allow programs to control and communicate with each other. For example, data collection can be initiated by a script with a resulting customized report automatically being sent to a printer, word process, spreadsheet, or LabVIEW for online process control.

These features greatly improve efficiency when combined with standard chromatography features like calibration factors, peak names, and graphical displays.

### Developing a chromatography user interface that adapts to a chemist's environment

*Mark W. Andrews and Monteith G. Heaton, Millipore Corporation, Waters Chromatography Division, 34 Maple Street, Milford, MA 01757*

Chromatography systems today are designed to provide specific specifications and mode of operation for software regardless of a chemist's environment. A project was undertaken to develop a system that can adapt to the way a chemist works in the laboratory. A review of the process by which a chemist obtains results was presented including discussions of the laboratory environment for Research, Methods Development, Quality Control/Testing and the interaction between these functions.

Several examples of adapting a user interface were shown with explanations of why these were done. Further examples demonstrated the ease of tracking and trending results using a database. Discussions also focused on GLP and regulatory compliance for standalone workstations and in a network environment.

### Computer-assisted methods for improving the quality of a comprehensive mass spectral library

*Stephen E. Stein, NIST Mass Spectrometry Data Centre, NIST, Gaithersburg, MD 20899*

A new strategy for building and maintaining a widely-used mass spectral library (the NIST/EPA/NIH Database) has been gradually implemented over the last four years. This was done in response to the 'maturation' of the library, which now contains spectra of a substantial fraction of those compounds of general analytical interest. Current emphasis is now on improving the quality and utility of the library by evaluating and (re)measuring mass spectra.

In the past, spectral quality was monitored by a computer-generated 'quality index' and new spectra came primarily from the mass spectrometry community in response to solicitations. However, since current computer methods cannot really evaluate mass spectra, this approach did not identify many erroneous and low quality spectra and even occasionally replaced an older higher quality spectrum with a newer one of lower quality. Further, the policy of growth by donation had little promise of providing spectra for most important compounds still missing from the library after 15 years of solicitation.

The current approach has assigned the primary responsibility for spectral quality to individual evaluators and the acquisition of new spectra to a directed measurement program. Instead of being used for quality measurement, computer methods now identify and assist the evaluation of suspect spectra. They also aid the selection of compounds for measurement and remeasurement. The discussion addressed failures of previous computer methods and provided details of the new methods. Topics discussed included: (1) the failure of the current 'quality index' to perceive certain classes of spectral errors; (2) method of finding suspect spectra and assisting their evaluation; (3) identifying groups of related spectra for evaluation; (4) perceiving 'important' compounds; (5) effects of spectral quality on library searching. Future methods that make use of chemical structures for locating errors and for estimating retention indices were also discussed.

### An effective chemical inventory system for the 90s

*Scott A. Jenkins, WillStein Software, Inc., 1723 Elmwood Ave., Wilmette, IL 60091*

In the chemical laboratory, chemical storage presents one of the largest potential threats to researchers, site, and surrounding communities. Incompatible chemical storage, overcrowd cabinets, infrequent inventories, and reordering of existing chemical stock are all problems that befall most organizations today. These problems could be resolved with an effective Chemical Inventory System (CIS).

Many CISs focus primarily on the safety issues, while others emphasize the ability to find and track the chemicals in the laboratory. The most effective chemical inventory should provide both of these functions in a user friendly environment. PCs and Bar Coding technology provide this type of environment.

Chemical Inventory and Tracking Essentials Professional (CITE Pro) provides crossplatform (Mac and PC) software and procedures for tracking chemicals throughout a scientific laboratory. Bar-codes are used to improve the efficiency of administration and to insure that all users always have access to accurate information.

Users have immediate access to the inventory from their desktop. Searches are performed very quickly by name, formula, location, or any other criteria. Each code is linked to a user-defined on-screen map showing the exact location (room and cabinet) for that chemical. Additional fields are provided to record amounts, hazards, disposal dates, or any other information. Report generation capabilities assist in the internal and external (SARA 313, tier I and tier II) auditing of the chemical site.

### The advantages and costs of using network backup solutions

*Brian E. Anthony, Dan Pearce and Doug McIntyre, Hewlett-Packard, Inc., Analytical Products Division, Palo Alto, CA 94034*

In the age of information the last thing the chemist needs is to be tied down by the task of providing adequate backups for the laboratory. While backups are seen as a task that must be done out of compliance with regulatory practices, backups also serve the necessary function of securing valuable data on which decisions are based that literally shape the world.

The process of backing up laboratory data is greatly influenced by the size of the laboratory. Needless to say, modern instrumentation produces larger and larger amounts of data in an effort to reach lower and lower sensitivity. These cumbersome volumes of data need to be managed just like any other task.

This paper used three examples in driving the point home of making backup work for the laboratory instead of the laboratory working to perform backups. Redundancy is necessary to safeguard against hardware failure and human error.

The first example explored was a client/server approach, which involves a large, multifaceted laboratory that needs a painless backup strategy that does not interrupt the laboratory production. This approach has very special needs that deal with schedule backups around the analyst. The second dealt with a medium-sized laboratory that utilizes optical diskettes, NFS, and magnetic tape to manage its backup needs. The last example stressed how a small laboratory not only needs central backup but also the flexibility to meet all regulatory requirements.

### Introduction of a fourth generation modular language for the control of instruments in the open systems environment

*Derek F. Rhodes, Peter Yendle, Kevin Smith and David Hurt,*

*VG Data Systems, Division of Fisons Instruments, 32 Commerce Center, Danvers, MA 01923*

VG Data Systems, a division of Fisons Instruments, has developed a novel and easy to use fourth generation language specifically for the application and control of chromatographic instruments in the chemical laboratory. In addition to allowing customization of control codes, the control language also allows complete customization of the user interface to invoke the parameters of choice.

The control language has been written in a modular form using object oriented sets of libraries and calling routines. These programming routines have been designed with an open systems approach such as to facilitate simple application of the control code to a variety of well known and not so well known instruments. This modular programming approach is referred to as Flexible Control Programming. Flexible Control is designed to work using the same command codes specified by the instrument manufacturer. In order to implement Flexible Control, applicable instruments must be amenable to communication via an industry standard communications port, i.e. adhere to open system standards.

Flexible control has been designed to be highly portable across a variety of platforms including VMS, ULTRIX, UNIX, DOS and the new ALPHA technology. This gives the user or programmer the opportunity to transport code across platforms or choose the platform most suited to their needs.

The advantages of control from a single point have been well documented. Probably the greatest benefit is compliance with GLP in having the security of single point information storage for the conditions of the chromatographic run along with the methodology and raw data files.

Flexible Control is designed to be a compiled language and as such executes very rapidly and is not accessible to unsupervised editing of parameters. Parameter definition, code structuring, compiling and implementation of this Flexible Control language was discussed. The Control code was presented with examples of real applications to the most common chromatographic instruments.

### A new instrument for voltammetry in flowing streams

*Peter E. Sturrock and Gerald E. O'Brien, School of Chemistry, Georgia Institute of Technology, Atlanta, GA 30332-0400*

A new instrument for voltammetric measurements in flow injection analysis (FIA), high-performance liquid chromatography (HPLC), and process control applications has been designed, constructed, and evaluated. The instrument uses conventional analog circuits with an imbedded 80156 microprocessor with 512 K RAM and 64 K EPROM. The instrument operates in conjunction with a host PC to which it is connected via an RS232 port. Operating programs, data files, and data-analysis programs are stored on the hard disk of the host PC. All timing for control and acquisition is provided by the 80186, programmed in an interrupt mode. Acquired data are stored in the instrument RAM by DMA and then transferred, a voltammetric sweep at a time, to the host PC which verifies the check sum, stores the data in a file, and displays the results in near real time.

One unique feature is the ability to change parameters during an experiment. This allows parameters to be optimized during a run and avoids having to abort the run and restart with new parameters. This is especially advantageous when using the instrument as a detector for HPLC where it might be necessary to delay a significant time for flushing the column before restarting the experiment. Each time parameters are changed, a new parameter set is stored in a file. Each sweep of data is identified by the parameter set used during its acquisition.

Experimental results obtained with the new instrument were presented and discussed.

### Monitoring of oxygenated additives in gasoline using the selective O-FID

*Ulrich K. Goekeler, ES Industries, 701 South Route 73, Berlin, NJ 08009-2621*

EPA regulations require monitoring the total amount of organic bound oxygen (oxygenated hydrocarbon contents), among other gasoline properties, of reformulated gasoline. Because the gasoline matrix consists of several hundred components, it is difficult to monitor a few oxygenates and impossible to monitor the total amount of organic bound oxygen. A selective detector, such as the O-FID, is capable of selectively detecting hydrocarbons which contain one or more oxygen atoms per molecule.

Principally, the analytical system consists of a manual, automatic, or an on-line split injector, and a capillary column. A cracking reactor, after the capillary column, disintegrates the molecules into their individual atoms and builds carbon monoxide, which is transferred into methane in the methanizer and detected with an FID. Therefore, for each oxygen atom in a molecule, one methane is generated.

At this point in time, the proposed ASTM method monitors 15 oxygenated hydrocarbons in a cycle time of approximately 21 minutes, and in a concentration range between 200 ppm and 20% for each component individually and disregards entirely the presence of hydrocarbons.

Practical examples for the determination of oxygenates in gasoline was discussed using chromatograms, the suggested EPA and proposed ASTM methods were explained, as well as the advantages and limitations (minimum sensitivity, linearity, cracking capacity, reactor lifetime), of the analytical system.

### Fingerprinting sulphur compounds in crude oil using gas chromatography with atomic emission detection

*Ronald D. Snelling, Institute for Environmental Studies, Louisiana State University, Baton Rouge, LA 70803 and William J. Wade,*

*Department of Geology and Geophysics, Louisiana State University, Baton Rouge, LA 70803*

The sulphur-containing heterocyclic compounds in a suite of Smackover Formation sourced crude oils were fingerprinted using gas chromatography with atomic emission detection (GC-AED) instead of the customary gas chromatography-mass spectrometry (GC-MS). The major advantages of this technique are simplified sample preparation and fewer interferences in the analysis.

The standard sample preparation of a sample for the analysis of the sulphur heterocycles involves fractionation of the oil using either column chromatography or HPLC. For analysis using GC-AED the only sample preparation required was treating oil diluted in cyclohexane with 5 A molecular sieves to remove the majority of straight chain alkanes.

After the straight chain alkanes were removed, there were no coeluting compounds which interfered with the peaks due to the sulphur heterocycles. Using GC-MS, there are coeluting compounds which give interfering peaks even if selected ion monitoring is used. GC-AED allows rapid, interference free fingerprinting of sulphur compounds in crude oil.

Variations in the concentration and distribution of sulphur containing heterocycles were found to be a function of thermal maturity of the oils. There were also significant differences between sweet and sour crude oils.

### The use of intelligent batch sequencing for increased productivity

*Kimberly A. Felt, Philippe Levi, L. Danny Hoey and Patrick D. Perkins, Hewlett-Packard, Scientific Instruments Division, 1601 California Avenue, Palo Alto, CA 94304*

Many laboratories have improved their operational efficiency and increased the accuracy and reproducibility of their data by going to automated GC/MS analysis for quantitation of target compounds. By using an autosampler and batch sequencing software the analyst is free to perform other tasks. However, until recently the analyst still had to go back at the end of the batch or sequence and reinject a sample or entire batch when quantitation results were outside of certain parameters.

With the introduction of more capable software which allows intelligent batch sequencing the analyst can now perform true automated analysis. This software exists for DOS based data systems that use HP GC/MS operating ChemStation software. Automatic evaluation, with user defined parameters, or data generated for each sample can decide on the next course of action. Simple tasks, such as injecting a blank between each sample, or deciding if a blank or subsequent sample is contaminated, can be easily added to any batch sequence. If a blank is contaminated the system can be made to cycle through a series of blanks until an uncontaminated blank is found or if not found, the system will continue or abort the sequence.

This work explained the typical decision criteria a user makes when evaluating a blank, negative, calibrator, control, or sample result. It was shown how this, user defined, criteria is easily incorporated into a sample method so that the system, independent of the user, can automatically make these decisions. Complex tasks such as deciding when to reinject a sample based upon whether retention times, ion ratios, or any other user selectable parameters is within acceptable limits can now be automated. A sample log table tracks any reinjections made by the systems intelligent sequencing. These and other examples were shown which illustrate how this unique capability has increased the productivity of laboratories involved in GC/MS testing of target compounds.

### On-line performance verification for a hygrometer in critical process monitoring applications

*J. Mettes, C. Haggerty and E. Zoladz, Meeco, Inc., 250 Titus Avenue, Warrington, PA 18976*

Even at low concentrations, moisture in semiconductor process gases has a negative impact on process yield. Hence, the detection limit of monitoring equipment is often taken with a large safety factor to assure detection. To ultimately safeguard a process, it is necessary to perform regular on-line sensor responsiveness checks for low level intrusions of moisture. A technique was presented that regularly adds small, well-controlled amounts of moisture to the sample gas and verifies automatically if the hygrometer's response is sufficient.
Regular additions keep the hygrometer slightly wet, which shortens response time when an intrusion actually occurs. A very important aspect of this technique when applied to the electrolytic hygrometer is that, despite such wetness, it still allows determination of moisture concentrations below the levels of the additions. To distinguish the contribution of the moisture in the sample gas from the added moisture, the instrument integrates the moisture measurement over five to ten-minute time periods at different flow rates. This approach enables lower detection limits because it negates flow independent background contributions.

Capabilities and limitations of this technique were discussed, along with examples of measurements down to 4 ppb generated by a calibrated standard.

### *In situ* sample preparation and optical analysis for process monitoring applications

*Ian L. Holwill and Nigel J. Titchener-Hooker, Advanced Centre for Biochemical Engineering, University College London, Torrington Place, London WC1E 7JE; Gareth B. Davies, B.R.F. International, Lyttel Hall, Coopers Hill Road, Nutfield, RH1 9HY, UK*

The optimization of purification processes downstream of fermentation systems producing intra- or extra-cellular products requires both rapid monitoring and control. High yields at each step in such multi-stage purification schemes are crucial to ensuring high overall yields. Process monitoring is used in order to maintain unit operations within required limits and provided measurement is fast enough appropriate action may be taken to hold the process within such limits and to optimize yield. However, in the case of biological processes retrospective correction must be made on a batch-to-batch timescale due to the intensive sample preparation required prior to analysis and the length of time this involves. This is particularly true of intracellular components which have to be released by cell disruption prior to measurement and hence may be associated with a large quantity of cellular debris.

A particular problem that is being studied is the detection of protein particles in the presence of yeast homogenate. Such particles are about 80 nm in diameter and are hidden amongst the larger sized yeast debris particles. This poses difficulties in interpreting light scattering data collected from the sample.

The authors described the development of techniques and equipment to overcome such difficulties and operate in an at-line environment.

### A multi-channel photometric detector for multi-component analysis in flow injection analysis

*Aimin Tan, Jinhua Xu, Jialin Huang, Liudi Geng and Xinna Zhao, Center for Process Analytical Chemistry, Department of Chemistry, Central South University of Technology, Changsha, Hunan 410083, P.R. China*

The conventional photometric detectors employed in flow injection analysis are so-called 'single-channel', which can equip only one flow cell. As a result, several individual detectors need to be used together in order to accomplish multi-component analysis, which makes the instrument set-up not only complicated and expensive, but also less reliable. Therefore, a single detector which can simultaneously monitor more than one flow cell is of clear advantages. In this paper, such a detector is designed by employing multi-wavelength LEDs and phototransistors for absorbance measurement with the control of an Intel 8031 8-bit single chip microcomputer. In the photometric detector, up to 4 flow cells can be attached, each of which is equipped with a multi-wavelength LED as the light sources and a silicon phototransistor as the photoelectric detector. By microcomputer control, all the LEDs are switched on and off sequentially, giving off different light beams. After being absorbed by the solutions in the flow cells, the light beams fall on the phototransistors. The current generated is then amplified and digitalized by a 12-bit A/D converter. Finally, the absorbances for each flow cells are calculated. As all the procedures are executed very quickly (compared with the speed of absorbance change), the flow cells can be monitored simultaneously. Because the other associated electronics are shared, and the LEDs and phototransistors are small, cheap, and reliable, the detector can also be made simple and reliable. As an application, such a detector is employed for simultaneous determination of trace amounts of cobalt and cadmium in zinc sulphate electrolyte, with two attached flow cells (one for cobalt, the other for cadmium). Because of the new multi-channel detector, this approach employs much less hardware apparatus than by employing conventional photometric detectors.

### Determination of gold in blood fractions by FIA and HPLC with ICP-MS detection

*Yafei Zhang, R. C. Elder, John G. Dorsey, Katherine Tepperman, Evelyn V. Hess and Keith G. Pryhuber, Biomedical Chemistry Research Center, Department of Chemistry, University of Cincinnati, Cincinnati, OH 45221-0172*

Gold drugs have been used for more than 60 years to treat rheumatoid arthritis without their metabolism being understood. Element specific monitoring of gold in body fluids can be used to study the metabolism and toxicity of gold-based drugs, but the low gold levels in patient blood or urine makes characterization of individual gold complexes very difficult.

This work described an analysis method developed to separate and determine the binding and levels of gold complexes in blood serum and red blood cells after incubation *in vitro* and also from patients undergoing chrysotherapy. Blood cells were separated by centrifugation. Possible gold metabolites were separated by HPLC after removal of proteins by ultra filtration or using direct sample injection onto an internal surface reversed phase column. An inductively coupled plasma mass spectrometer (ICP-MS) was used as a detector, giving a very low detection limit, to selectively monitor the separated Au-containing species such as $[Au(CN)_2]^-$ (an important metabolite), the complexes with cysteine, glutathione or the gold drugs themselves. The mobile phase used in HPLC-ICP-MS system contained methanol, tetrabutylammonium chloride (TBAC—as an ion-pairing agent) and phosphate buffer solution (pH = 7.4). Effects of various mobile phase parameters were investigated. It was found that $[Au(CN)_2]^-$ entered red blood cells rapidly when they were incubated with $[Au(CN)_2]^-$. About 90% of total gold was found in red blood cell lysate, 6% in the serum fraction and 4% was bound to the membranes. Hemoglobin appeared to be the main species binding gold in red blood cells. Different patients had very different gold level in their red blood cells. The distribution of gold in red blood cells seemed to correspond closely with the distribution of sulfhydryl groups. Most of the gold in the patient blood was bound to serum albumin. This method is very sensitive and can be used to detect gold-containing species in blood or urine samples.

### Near-infrared FT-Raman spectrometry as a tool for quality control

*R. A. Spragg, C. M. Deeley and J. Sellors, Perkin-Elmer Ltd, Post Office Lane, Beaconsfield, Buckinghamshire HP9 1QA, UK*

Raman spectrometry has the potential to be an excellent tool for QC. The spectra can provide as much qualitative information as IR spectra can, but without any sample preparation. However, until the development of Fourier transform Raman spectrometers the sample turnaround rate was too slow to make this a realistic possibility.

The suitability of NIR FT Raman spectrometry for rapid verification of the identity of a variety of materials has been investigated. The major issues addressed were the ease of presentation of different types of sample, the speed of measurement, and the choic of algorithm for spectral comparison.

The speed of measurement is an issue because in a given time the signal-to-noise ratio of Raman measurements is significantly lower than for FTIR. Fortunately the NIR FT-Raman technique has developed to the point where 30 seconds measurement using a room temperature InGaAs detector is often sufficient. However, high frequency noise which can be reduced by extended signal averaging is not the only problem. Variations in sample position can cause spectral changes in all types of sample. For solids there are additional sample-to-sample differences which typically appear as broad baseline features, while sample inhomogeneity can be another source of variation.

Two approaches to overcoming these difficulties were considered. The first is to filter the data in order to discriminate in favour of the spectral information and against both broad baseline features and noise. The second is to use a modelling approach based on principal components analysis so that any expected spectral variations are allowed for in the spectral matching process. As the two approaches address different problems they can usefully be combined.

### Application of Raman spectroscopy to on-line process control and surface contamination monitoring

*Cathy D. Newman, John D. Algeo, Georges G. Bret and Kerry M. Swift, Chromex, Inc., 2705-B Pan American Fwy., NE, Albuquerque, NM 87107 and Thomas M. Niemczyk, University of New Mexico, Department of Chemistry, Albuquerque, NM 87131*

CCD Raman spectroscopy has new promise as a tool for supplying detailed and useful information for monitoring and controlling chemical processes. Advanced, simplified instrumentation is now available which can acquire data quickly and continuously. Its enhanced sensitivity with diode laser excitation in the near IR allows the use of optical fibres as in-line probes, transmitting the laser excitation and collecting the Raman scatter. Fibre optic probes isolates laboratory-grade spectroscopic instruments from harsh conditions in the plant.

New results were presented for samples representative of chemical processes, including some with special challenges. The samples included organic and inorganic chemicals in polar and non-polar solvents, slurries, and solids. For example, chemometric methods were applied to determine actinide metals in highly concentrated nitric acid solutions such as those used in nuclear fuel reprocessing. Excellent correlation was found for concentrations calculated from Raman spectroscopic data.

### New instrumental developments in near-infrared spectroscopy and their applications in chemical quality- and process-control

*U. Eschenauer, M. Hühne, J. Hildebrandt, I. Zebger and H. W. Siesler, Department of Physical Chemistry, University of Essen, D-4300 Essen, Germany*

Despite its lack in structural interpretative value, near-infrared spectroscopy is increasingly accepted as a powerful technique for chemical quality assurance and process-control. This acceptance is further enhanced by the current development of new sensors and rapid-scan near-infrared monochromator/detection systems without mechanically moved parts, such as Acousto-Optic Tunable Filters (AOTF) or diode arrays. In combination with optical light fibres, the resulting miniaturization of near-infrared spectrometers greatly facilitates their implementation in hostile plant environments.

This contribution focused on selected examples of rapid identity control of liquid or solid materials (for example for industrial chemical raw materials and with reference to recent work in polymer recycling). In the second part, examples of reaction control and quantitative determination of the composition of liquid process streams with specific sensors (for example by suppressing the interference of solid particles in suspensions) was addressed.

### Ultra fast full spectrum analyser for process applications

*Isaac Landa, L T Industries, Inc., 6110 Executive Blvd., Rockville, MD 20851, Luis M. Dominquez, R J Reynolds Tobacco Company, Winston-Salem, NC 27102*

A novel fast scanning NIR analyser for reflectance or transmittance measurement capable of over 100 real time multi-variate analysis per second has been developed. The analyser consists of an eight channel single fibre optics multiplexer, an in-line wavelength reference and integrated source. The system is driven and enhanced by Chemometrics software package utilizing DMA, DSP and Windows$^{TM}$ capabilities. Additionally, the analyser is configured to meet NEMA 4X industrial requirements. Process capabilities were described and limits of performance presented.

### Automated stepwise isothermal thermogravimetric analysis

*William Sichina, Seiko Instruments USA, 111 Gibraltar Road, Horsham, PA 19044*

Resolution of successive thermogravimetric (TG) decomposition events is most effectively achieved using an automated isothermal stepwise approach. This entails heating a sample at a constant rate until a significant weight loss occurs, as determined by a threshold level based on the rate of mass loss (μg/min). The TG instrument automatically holds the sample under isothermal conditions until the rate of decomposition decreases to the point where it becomes less than the exit threshold rate value. The instrument then automatically resumes heating at a constant rate until the next significant mass loss event is encountered.

The automated stepwise isothermal approach was used to successfully characterize the decomposition properties of three polymeric materials: ABS (acrylonitrile-butadiene-styrene blend), 'plastic wood' blend, and an elastomer. The results obtained using the auto stepwise isothermal technique were directly compared to conventional, constant heating rate methods.

For the ABS material, it was found that the blend yields two well-resolved decomposition events using the auto stepwise approach: the decomposition of the SAN copolymer continuous matrix at 390°C and the decomposition of the rubber component at 445°C. The results obtained via the auto stepwise technique are displayed in the figure below for the ABS blend. It was found that two different ABS blends (which were reported to have different toughness characteristics) had significantly different compositions as determined using this technique. It was found that the three major components (oil, polymer and carbon black) comprising elastomeric materials could be successfully and reproducibly separated using the auto stepwise approach. It has been difficult to separate the volatilization of the oil from the decomposition of the polymer using standard, constant heating rate TG experiments.

### A robotic system for complex sample preparation controlled by high level scheduling and tracking algorithms

*Steven D. Hamilton, Nader Donzel, Jean-Marc Haab, Frederic Mottier, Jorge Carmona, Reto Buhlmann and Jose Gil, Scitec Consultants, Inc., 105 Barksdale Professional Center, Newark, DE 19711*

A robotic system to perform complex biological sample preparations has been developed by Scitec using the Hewlett-Packard Optimized Robot for Chemical Analysis (ORCA). This system performs operations such as capping/uncapping tubes, automated GC/LC vial crimp capping, bar code labeling and reading, liquid/liquid extractions, liquid phase transfer, liquid dispensing and controlled temperature incubations. A high level software package, CLARA (Computer Logic Applied to Robotic Applications), has been developed to design, create and optimize a scheduling template for an application in compliance with robotic resource and sample processing time constraints. This template is incorporated into CLARA Kernel (a subset of CLARA), which provides a user interface while running, monitoring and logging all operations of the robotic system. This application is designed to meet challenging GLP requirements via features such as user sample identification, bar code label application and logging of timed events and errors in appropriate files.

### Constrained learning algorithms for backpropagation neural networks

*Peter de B. Harrington, Ohio University, Center for Intelligent Chemical Instrumentation, Department of Chemistry, Clippinger Laboratories, Athens, OH 45701-2979*

Neural networks are computer systems that simulate biological nervous systems. Backpropagation neural networks (BNNs) have generated much interest in analytical chemistry because they are powerful pattern recognizers. Neural networks were designed to be implemented in massively parallel configurations. However, most chemical applications use minimal network configurations, i.e. layers and units. The reason for this parsimonious approach is that neural networks such as backpropagation and counterpropatation are trained by minimizing prediction error. Often the networks are deleteriously pared down to avoid overfitting the data. In addition, determining the minimal configuration is problematic.

Delta learning minimizes prediction error in an unconstrained manner. By constraining the algorithm that distance in the data space becomes important, the modeling of neural network may be softer. Therefore overfitting may be controlled in large parallel network architectures. Normalized unit weight vectors and a temperature parameter in the logistic function, allow BNNs to train or optimize in an analogous fashion to simulated annealing. This approach is successful with minimal neural networks that use localized processing.

An algorithm that accurately propagates errors back through normalized weight vectors during training was presented. Global and local temperature controlled studies were evaluated. These neural networks have the advantage that high resolution data can be processed directly, instead of imposing data compression methods such as principal component analysis.

### Optimization of neural networks configurations by experimental design

*Peter J. Tandler and Peter de B. Harrington, Ohio University, Center for Intelligent Chemical Instrumentation, Department of Chemistry, Clippinger Laboratories, Athens, OH 45701-2979*

The backpropagation neural network (BNN) is a powerful chemometric tool. The determination of the number of nodes and layers within the network is problematic. Methods for minimizing the size of the network have been devised, but there is some disagreement on the use of the minimum number of nodes.

Minimally configured networks are dependent on the initialization parameters and are more susceptible to local minimum. Increasing the number of nodes usually decreases the number of training cycles, but a loss of generalization may be obtained. Generalization is the ability to make accurate predictions with data similar but not identical to that used in training. The effect of BNN configuration is dependent on the data set and, to complicate matters, the selection and the size of the training set. The latter has received recent attention.

In addition, generalization may be improved by early termination of training. Deciding when to stop training is difficult, and is usually accomplished with a second evaluation set. BNN performance is affected by training set characteristics (for example size and scope), network configuration and training duration. These factors are mutually dependent and are evaluated using a factorial analysis. Optimal training set attributes are exemplified with various simulated and mass spectral data sets.

### Characteristics of a method for the determination of kinetic parameters for consecutive first-order reactions using the Kalman filter

*Min Gui and Sarah C. Rutan, Department of Chemistry, Virginia Commonwealth University, Richmond, VA 23284-2006*

Many analytical reagents react with analytes according to a rate law described by consecutive first-order reactions—a product formation step, followed by a product degradation step. In this work, factors that affect the determination of the kinetic parameters with the Kalman filter for this class of reactions have been investigated by computer simulation. The effect of changing the rate constants, the initial concentration of analyte, the fitting range, the date density, the mismatch between the measured time and real time, and the initial estimate for the background signal has been evaluated by using synthetic data with Gaussian distributed noise. The percent errors in the estimated rate constants and the initial concentration of analyte have been evaluated for each of the different variables under consideration. Plots of these errors as a function of the various effects mentioned above permit the method to be completely characterized. Empirical methods for obtaining initial estimates for the rate constants and the initial concentration from the original kinetic data, based on the model for consecutive first-order reactions, have been developed. An algorithm has been proposed and a program has been written which gives satisfactory fitting results for an unknown sample whose rate constants fall in the range of 0.05 to 1 $min^{-1}$.

The method was applied to experimental kinetic data based on the reaction of amino acids with the fluorogenic reagent, *o*-phthalaldehyde, after thin-layer chromatographic separation. The experimental kinetic data were extracted from the images of the chromatographic plate, taken at regular time intervals with a charge-coupled device (CCD) camera.

### Applications of butterfly neural network for non-linear principal component analysis

*Busolo Wa Wabuyele and Peter de B. Harrington, Ohio University, Center for Intelligent Chemical Instrumentation, Department of Chemistry, Clippinger Laboratories, Athens, OH 45701-2979*

The volume of data generated by analytical instruments has increased tremendously over the past two decades. Most of the data produced by modern analytical instruments is multivariate in nature and produced over a small time. The objective is to carefully extract and interpret relevant analytical information. Computerized data analysis has now become a practical tool in data manipulation.

Principal component analysis (PCA) is a useful tool for reducing the dimensionality of multivariate data. PCA compresses information in a data set by generating linear combinations of the variables that maximize variance. PCA has broad application that includes calibration, exploratory analysis, classification and feature selection.

Nonlinear PCA may be obtained by butterfly neural networks. These systems are backpropagation neural networks that are autoassociative (i.e. use the same data for both input and output). By reducing the number of hidden units the information content stored by these units will be maximized in an analogous manner to PCA. These networks have a shape of the butterfly, hence the name. The outputs from the hidden layer units are analogous to observation scores generated during PCA. This technique will be evaluated for non-linear modeling of infrared and visible spectra.

### Development of digital filtering techniques as preprocessing tools for multivatiate calibration

*Gary W. Small, Center for Intelligent Chemical Instrumentation, Department of Chemistry, Clippinger Laboratories, Ohio University, Athens, OH 45701-2979*

Current state-of-the-art procedures for implementing spectroscopic-based quantitative analyses focus on the use of multivariate calibration models based on spectral variables obtained by applying principal components analysis (PCA) or partial least-squares (PLS) analysis to a set of calibration spectra. Multiple linear regression analysis is subsequently used to correlate known concentrations to the spectral variables. The PCA and PLS procedures are useful largely because they allow spectral information pertaining to the analyte to be extracted from that due to interferences or spectral noise. The PLS technique is particularly powerful in that it employs the known concentrations of the calibration samples to extract spectral information that correlates with analyte concentration.

A limitation of the PCA and PLS techniques is that they can be confounded by spectral baseline variations or by noise features that provide chance correlations with concentration. It is therefore advantageous to preprocess the spectral data to remove these effects.

The author's laboratory is currently evaluating the utility of digital filtering techniques as a generalized spectral preprocessing tool. Filtered spectra are subsequently submitted to PCA or PLS and the calibration model is computed as usual. In the work presented, the design of the digital filters used was explored. Utilizing analysis of variance techniques, the parameters used in the filter design were examined and a general protocol for the design was established.

### A SVD-based efficient numerical algorithm for performing partial least squares (PLS)

*T. W. Wang, M. Berry[†], J. Batra, A. Khettry, P. Sarma and M. Hansen, Department of Chemical Engineering, [†]Department*

*of Computer Science, The University of Tennessee, Knoxville, TN 37996-2200*

The use of multivariate statistical techniques, such as principal component regression (PCR) and partial least squares (PLS), has become increasingly popular in the field of chemometrics and process monitoring, because of the ability of these approaches in handling collinearity, and noise.

In essence, the approaches of PCR and PLS are based on extracting a much smaller dimensional model in representing the original array of process data. Then, the original data are projected onto this reduced dimensional subspace followed by linear regression carried out in this subspace. Finally, the regression is transformed back to the original and larger dimensional space. PCR and PLS differ in the way the original data is reduced to the smaller subspace in that in PCR, only the input space data, the $X$ matrix is used in the dimension reduction step; whereas PLS uses both the $X$ as well as the output data matrix $Y$ in doing the space reduction. The rationale of PLS is that, in doing PCR, it may occur that a small yet significantly correlated to the output $Y$ information may be discarded during the space reduction step. Therefore, in PLS, the reduced data space is optimally correlated to both $X$ and $Y$ simultaneously, but not optimally correlated to either $X$ or $Y$ alone.

Traditionally, in the numerical approach for both PCR and the PLS, the power method of NIPALS (nonlinear iterative partial least squares) has been used in which iteration is involved in calculating each of the principal loading and score vectors. One drawback with the NIPALS approach is its relatively slow computational speed. An approach based on singular value decomposition (SVD) of the appropriate matrices would overcome both of these drawbacks.

A SVD-based approach is proposed for performing PLS. The sample data size used was a 500 × 2, . . . , 5) output $Y$ and a 500 × 20 input $X$ matrix respectively. The procedure was to first form the covariance matrix $X^TY$. Then the principal component that would account for the largest variation of $X^TY$ was found through the use of SVD. This is equivalent to finding the largest singular value and the associated left singular vector of $X^TY$ made by the first principal component. Then the process repeats with the new residual. The process is terminated when the number of factors deemed having significant contribution to the variation of $X^TY$ has been reached. This can be arrived at by cross-validation test. In finding the SVD of the residual matrix $X^TY$ in each round of factors, the so-called economy SVD routine is employed. Since the size of $X^TY$ is rectangular $m \times n$ with more rows than columns usually, then only the first $r$ left and right singular vectors are computed where $r$ is the rank. This gives a substantial saving over the full SVD in which all $m$ left and $n$ right singular vectors are determined.

PLS was performed using the SVD-based approach in Matlab implementation environment. Computational requirement based on traditional NIPALS method was used as the reference. A reduction by a factor of ten of the computational steps was realized with this SVD-based approach for this sample size. Preliminary work has also been carried out in using a Lanczos algorithm for approximating only the largest singular value and its corresponding left and right singular vectors at each step. This is applicable since only the largest singular value and its associated left singular vector are needed at each step. Preliminary results indicate that an additional saving of a factor of at least 3 can be achieved by this approach.

The SVD-based PLS approach has been applied in the in-line analysis of near infrared (NIR) monitoring of the changing composition in polymeric processes. Compared to a commercial PLS software based on NIPALS, the SVD-based approach rendered a somewhat smaller prediction residuals with an improved and more stable computational efficiency. Detailed procedures and case studies will be published elsewhere.

### Advances in machine learning of fuzzy rules as applied to spectroscopic techniques

*Beata Walczak, Eva Wolf-Bauer and Wolfhard Wegscheider, Institute for Analytical Chemistry, Micro- and Radiochemistry, Graz University of Technology, Technikerstrasse 4, A-8010 Graz, Austria*

Classical applications of neural nets have shown serious shortcomings in obtaining unique solutions in data reduction unless great care is taken to avoid nets that are larger than absolutely necessary. In the practice of analytical chemistry such minimal nets can be combined with bilinear methods, such as PCA and PLS, to give superior performance in multivariate calibration. These minimal nets that frequently have but one hidden unit in a single hidden layer are notoriously difficult to interpret and this is true whether they perform well or not.

This prompted the authors to investigate the performance of fuzzy neural networks. The conceptual advantage of these nets is their ready interpretability, as all the weights can be understood after training as the relative importance of a fuzzy logic rule. These fuzzy rules are combined by various logic operators, such as OR and AND, and even extension of these. This view of a neural network as a complex decision tree combines the advantages of machine learning from examples with the interpretation power of fuzzy logic that in earlier work has shown to perform well on the frequently vague data derived from spectroscopic techniques.

Aiming at the best of both techniques it was shown how neural network training can be applied to the learning of the importance of rules and of membership functions that are optimal with respect to maximum discrimination between constituents in mixtures. This was shown for the case of X-ray fluorescence spectra taken by wavelength dispersive spectrometry.

### An expert system for polymer characterization

*David J. Ramsbottom, Michael J. Adams, School of Applied Sciences, University of Wolverhampton, Wulfruna St., Wolverhampton, WV1 1SB, UK; John Carroll, I.C.I. Wilton Materials*

*Research Centre, P.O. Box 90, Wilton, Middlesbrough, TS6 8JE, UK*

Expert systems are one of the most rapidly developing areas of applied artificial intelligence, and in recent years the availability of sophisticated expert system shells has further increased interest in their use in analytical laboratories. One factor which can have a profound effect on the efficiency and efficacy of expert systems is the means of generating and propagating uncertainty throughout the system. Use of the most appropriate uncertainty method often involves its implementation in software external to the shell and requires careful testing and evaluation.

The results reported here referred to a PC-based expert system developed to serve as an aid in the characterization of polymer samples. In a similar manner to the polymer analyst, the system uses a variety of physical attributes and the results of performing simple physico-chemical tests. Such tests, whilst subjective, provide a means of producing a short list of likely polymers and directing further strategies for the identification of additives. If an infrared spectrum of the sample is available, then interrogation of the spectral data can provide a more specific identification.

The results of implementing several uncertainty representations will be presented, including Bayesian, certainty factors and fuzzy logic. In practice, fuzzy logic techniques have proved the most useful in providing an effective, flexible analysis scheme.

A fuzzy c-means (FCM) pattern recognition algorithm has been successfully used to characterize polymers by their infrared spectrum. The results of the FCM provide the expert system with a value for the membership of the sample to each cluster of known polymers used in the training set.

The use of uncertainty in the expert system, and in particular fuzzy set theory, allows subjective tests to be incorporated successfully within the system.

The data analysis strategy, generation and propagation of uncertainty as well as the performance of the system were discussed.

### Simultaneous multi-element atomic absorption spectrometry

*Kimberly S. Farah and Joseph Sneddon, Department of Chemistry, University of Massachusetts-Lowell, Lowell, MA 01854*

Flame and furnace AAS are techniques widely used for the determination of trace and ultra-trace metals. One drawback of AAS has been its lack of ability to determine simultaneously several elements. In the past few years, several commercial systems have become available which allow for simultaneous analysis. The spectrometer used in this work allows for simultaneous analysis of up to four elements at a time. The system is based on one photomultiplier tube and uses a galvanometer driven grating in its monochromator. This mechanism allows the instrument to scan the full spectrum in 20 milliseconds. A galvanometer driven motor is used to select lamps.

This paper described the use and application of this simultaneous system. Optimization of the system for multi-element flame determinations was discussed. The results of varying the following parameters: air to fuel ratio, hollow cathode lamp currents, slit width, and height above the burner head, on flame sensitivity will be shown. Application of multi-element flame analysis for the determination of metals in soils was presented.

Multi-element analysis using the graphite furnace was discussed, including the use and advantages of the Smith-Hieftje background correction system. A comparison of the Smith-Hieftje and deuterium arc background correction systems for representative multi-element analysis was made.

### Simultaneous multielement atomic absorption spectroscopy with a nitrous oxide flame source and diode array detection system

*Clifton P. Calloway, Jr. and Bradley T. Jones, Department of Chemistry, Wake Forest University, Winston-Salem, NC 27109*

The major drawback to conventional atomic absorption spectroscopy (AAS) is that it is an inherently single element technique. While many detection systems, such as linear photodiode arrays (PDA), charge injection devices (CID), charge coupled devices (CCD), and photomultiplier tube banks (PMT), are capable of performing simultaneous multielement analysis, few multielement sources have been developed. Multielement hollow cathode lamps are currently available, but generally suffer from loss of intensity and only limited combinations are available. Continuum sources have also been used for AAS measurements, but generally exhibit smaller calibration sensitivities and linear dynamic ranges. This presentation described and evaluated the use of a nitrous oxide/acetylene flame emission source in which a mixture of the metals to be determined is aspirated. This source signal is focused through a second sample flame or graphite furnace, where absorption may occur. The unabsorbed radiant flux is focused onto the entrance slit of a high resolution (1.33 m) monochromator equipped with a 5 cm long, 2048 element, linear photodiode array detection system. Figures of merit for various elements and spectral windows were presented. With this system, an internal standard may be added to the source flame to correct for flicker noise in the flame and thereby enhance signal-to-noise ratios. This system provides quick, convenient method for switching analytes as well as multielement detection resulting in minimal down time between analyses. Results were given for both flame and furnace atomization cells.

### NetCDF software and tools, the *de facto* public-domain standard for data transfer, storage, and archival for analytical chemistry

*Rich Lysakowski, Digital Equipment Corporation, Four Results Way, MR04-2/C17, Marlboro, MA 01752*

Over the past three years, a public-domain system for scientific data interchange, storage, and archival has emerged as the *de facto* standard. This system is called the network Common Data Form (netCDF). NetCDF is a portable binary file system with an abstract software interface for data access. It was designed for large, multidimensional, scientific datasets. NetCDF was developed and is supported by professional scientific software engineers as the Unidata Program Center in Boulder, Colorado. The Unidata Program Center is federally-funded by the National Science Foundation. NetCDF has been used successfully for datasets in scientific visualization, meteorology, space science, computational fluid dynamics, chromatography, mass spectrometry, nuclear magnetic resonance, infrared, and UV-VIS spectroscopy.

NetCDF includes tools for converting binary datasets to human-readable representations, converters for converting Common Data Language (ASCII) datasets to binary (netCDF) datasets, and code generators for helping programmers create netCDF data conversion programs. Other tools that work on netCDF standard datasets are available, such as extractors for extracting subsets from large, multidimensional data files, operator for doing statistics on data within the files, and other operators. MS-Windows Dynamic Link Libraries (DLLs) are being developed that will be put into the public-domain to help create language-independent standard data access tools.

The major manufacturers of chromatography and mass spectrometry instruments and software have adopted netCDF along with a subset of the ADISS Analytical Information Model (ADISS AIM) as the public-domain standard for analytical data interchange and storage. In addition, large end user companies such as Kodak, American Cyanamid, Exxon, DuPont, Sandoz, and other have begun using the combined ADISS/netCDF standards for infrared, nuclear magnetic resonance, and UV-VIS spectroscopies. The ADISS/netCDF standards are now being applied to atomic absorption, atomic emission, thermal analysis, X-ray, and surface chemical analysis data.

This paper covered the design, technical features, and benefits of the netCDF software and its tools, a discussion of system performance, and examples of its application to analytical chemistry and spectroscopy.

### The analytical instruments associations standards program: applications

*David C. Nelson, Nelson Consulting, 855 Hamilton Ave., Palo Alto, CA 95301; Richard Lysakowski, Digital Equipment Corporation, 4 Results Way, MR04-2/C17, Marlboro, MA 01752 and Michael Duff, Analytical Instruments Association, 225 Reinekers Lane, Suite 625, Alexandria, VA 22314*

The Analytical Instruments Association (AIA) is a trade association composed of approximately 65 of the major analytical instrument suppliers.

The AIA has developed a Chromatography Data Communications Standard (reported on during the '92 Pittsburgh Conference). That Standard was issued in May 1992 and is available through the AIA.

This paper reviewed the basic concepts behind the standard, but emphasized applications implemented by actual users.

The Standard is implemented in two ways:

(1) By chromatography data systems suppliers who make the standard part of their product.
(2) By users who, themselves, perform the work to make the standard part of their systems activities.

Technical experiences of users and suppliers were presented, including problems encountered in the implementation that require revision consideration.

Future activities of the AIA programme, including revision plans and other analytical techniques to be pursued, were presented.

### Evolution of an interchange specification for mass spectrometric data

*David Stranz and Scott Campbell, Fisons Instruments, 809 Sylvan Avenue, Suite 102, Modesto, CA 95350; Ray Christopher, Finnigan MAT, 355 River Oaks Parkway, San Jose, CA 95134; Jim Watt, P-E Nelson, 10040 Bubb Road, Cupertino, CA 95014; and Don Zakett, Dow Chemical Company, Analytical Labs Building 1897, Midland, MI 48657*

The first version of the Analytical Instrument Association (AIA) Interchange Specification for Mass Spectrometric Data has been released. This specification has two components: a model which describes the information content of a mass spectrometric experimental data set, and an implementation of the model in portable source code utilizing a public-domain programming toolkit. Vendors of mass spectrometric hardware and software are now introducing software which produces interchange format files from vendor-specific data sets.

Numerous factors were key to the successful development of this specification: early commitment by the major vendors to co-operate in the effort; a broadly-based review committee drawn from manufacturer and end user communities; external resources, including the Analytical Instrument Association, the ADISS Project, and the Unidata Program Center; support from the ASMS; and a dedicated technical working group of champions.

This paper presented the history and current status of the AIA mass spectral data interchange specification, early experience with vendor implementations, and on-going efforts to create a more comprehensive model.

The AIA Interchange Specification for Mass Spectrometric Data has been placed in the public domain. The distribution kit may be obtained by contacting the Analytical Instrument Association, 225 Reinekers Lane, Suite 625, Alexandria, VA 22314.

### A status report on the Laboratory Automation Standards Foundation

*Joseph G. Liscouski, Laboratory Automation Standards Foundation, P.O. Box 38, Groton, MA 01450*

The case for data format and communications standards in laboratory computing has been made. The need now moves to ensuring that the existing and proposed standards are supported, and that new standards definition efforts are applied to significant problems.

This presentation was a status report on the activities of the Laboratory Automation Standards Foundation. The LASF has divided its activities into three areas: standards, the development of a discipline of Laboratory Automation Engineering, and technology for automation.

The LASF model for laboratory computing was reviewed. The purpose of the LASF model is to provide an implementation independent approach to automating laboratory work. It places emphasis on the use and flow of data and information in the laboratory, and the use of lab results by outside organizations. The result is a basis for identifying missing technologies, requirements for standards definition, and deriving flexible implementation approaches.

A proposal for the development of Data Librarian for the management of data modules conforming to instrument data standards was presented. The need for the librarian is a result of the LASF model.

The LASF has begun giving courses in conjunction with Worcester Polytechnical Institutes Continuing Education Department and other groups. The structure of those courses, their goals, and future direction were covered.

### Laboratory automation and method standardization

*Scott B. Tilden, Laboratory Expertise Center, Digital Equipment Corporation, 318 S. Main St., Albion, NY 14411*

The goal of the instrument interface in an automated laboratory is to transfer 'needed' data from the instrument to a laboratory information database such as a LIMS system. The data that is transferred from the instrument to the laboratory database is prescribed within the context of the laboratory 'method'. Few industrial method documents or method management strategies, however, were conceived with an eye to the challenges of automating instrument data collection. Few laboratories for example have method management strategies that define what is contained within a method document to address all the elements required to rigorously specify both the data and process that would be utilized in a totally automated instrument interface environment. Some of the critical attributes that must be rigorously defined by the method document include the actual format of the results data to be transferred; how the data is validated; what supporting data (such as operator ID or instrument condition information) should be transferred with the primary instrument results; and how the data is further reduced before the data is sent to the laboratory database. The lack of industry standardization of the critical elements that should make up a method document and methods management strategy has resulted in instrument interface programs that are costly and inflexible.

Though experience in creating instrument interface programs within large industrial laboratories, a beginning to creation of the required definitions and elements of an effective laboratory methods strategy is starting to coalesce. This strategy encompasses these method management elements:

(1) Definition of Method Hierarchies;
(2) Attributes of Methods at each Method Hierarchy;
(3) Consistency of Method Attributes Mapped to Major Analytical Technologies;
(4) Definition of Validation (and other) Processes Mapped to Major Analytical Technologies (Work Cell Automation).

Each of these methods management elements was briefly described in the presentation along with 'straw person' definitions and examples. Standardization of these method management elements and definition of critical attributes of these elements will allow interfaces to be created that address the following interface goals (1) low cost; (2) vendor supported; (3) flexible; (4) and able to survive audit scrutiny (GLP, GMP, GALP, FDA, ISO 9000, etc.).

### Exchanging data between computers is not easy. Are you brave enough to try?

*Richard D. Weimar, Jr., Competitive Edge, 514 Ivydale Rd., Wilmington, DE 19803-4330*

For data and information to be sharable between computer systems and to have value to the business, certain principles must be addressed:

- The sender and the receiver must have the same understanding of data and information shared
- People infer!
- Computers do not infer!
- Most computers are absolutely, rigidly logical.

Our not directly addressing these four principles is the fundamental cause for our not being able to effectively exchange data and information between absolutely, rigidly logical computers and information systems.

This paper presented a set of Critical Success Factors for developing Data Architectures which, when satisfied, allow these rigidly logical systems to share data and information effectively. Also presented was a process to develop and implement such an architecture.

### High speed, high sensitivity field screening of volatile organic compounds with a micro gas chromatograph

*Mark W. Bruns, Kent Hammarstrand and Chi K. Lo, MTI Analytical Instruments™, Microsensor Technology, Inc., 41762 Christy Street, Fremont, CA 94538*

Field screening for low levels of volatile organic compounds (VOCs) will become increasingly valuable as enactment of environmental regulations continues worldwide. Recently enacted environmental mandates press the need for rapid, rugged, reliable, high sensitivity instrumentation which can be utilized in the field for screening and monitoring purposes.

A low cost, high throughput, portable sample concentrator and gas chromatography system was developed for monitoring VOC contamination in air, water and soil. The battery-operated sample concentrator consists of a two sorbent trap system offering greater than 8 hours' continuous operation in the field sampling water, soil, or air (directly or from containers) with optional internal standard addition to each sample concentration event. The portable gas chromatograph features the use of capillary columns with a silicon micromachined injection and detection system, providing high speed, high resolution analyses in the field. The combined result is a field portable package capable of high sample throughput at part-per-billion level sensitivity for VOCs without the need of cryogenics, large power reserves, or canister based sampling; ideal features for an environmental field screening analytical instrument.

This presentation highlighted the system's performance relative to concentrating and analysing VOCs commonly found in air, soil, and water. The presentation emphasized the system's sensitivity and high sample throughput capabilities. The repeatability, calibration, continuous operation lifetime, and accuracy of the system were discussed.

### Rapid monitoring of transformer gases by high speed gas chromatography

*Kent Hammarstrand, Mark Bruns, MTI Analytical Instruments™, Microsensor Technology, Inc., 41762 Christy Street, Fremont, CA 94538, and C. Clair Claiborne, ABB Power Transmission and Distribution Company, Inc., Transmission Technology Institute, Centennial Campus, 1021 Main Campus Drive, Raleigh, NC 27606*

Gases such as hydrogen, carbon monoxide, carbon dioxide, ethylene and acetylene are key gases that will indicate incipient faults in power transformers. These gases together with other constituents are soluble in the insulating oil and can be extracted and analysed by gas chromatography.

The gases are separated from the oil insulation by a gas permeation cell. This gas permeation cell is located on the side of the transformer and oil is circulated into the cell by use of a pump or by a thermosiphon accessory which enhances the oil flow. The gases pass from the oil through a semi-permeable polymeric membrane protected by a fritted stainless steel support piece into a collection cavity. A few microlitres of gas from this collection cavity are then passed onto the gas chromatograph for an ultrafast analysis of the key gases.

Data was collected from a three column high speed gas chromatograph with a solid state detector, where the analysis time is reduced to seconds instead of minutes, unlike conventional gas chromatographs. The instrument is portable or can be utilized in the laboratory for fast, reliable analyses of fault gases at the ppm levels. The gas sample is injected onto the three columns simultaneously, and the analysis takes place in parallel with a data system reporting the gas composition. Faults such as overheating, corona discharge, sparking or arcing of the oil or solid insulation were verified with chromatograms and statistical data. Microbore capillary column technology and micromachined components are integral parts of the total system for high speed gas chromatographic analyses.

### Automated modeling techniques for $^{13}C$ NMR chemical shift prediction

*Robert C. Schweitzer and Gary W. Small, Center for Intelligent Chemical Instrumentation, Department of Chemistry, Clippinger Laboratories, Ohio University, Athens, OH 45701-2979*

A useful tool in the interpretation of carbon-13 nuclear magnetic resonance ($^{13}C$ NMR) spectral data is the ability to predict the chemical shifts of carbon atoms for which experimentally determined shifts are not available. Two practical approaches exist for estimating the chemical shift of a carbon atom. One method uses database retrieval techniques. The database is composed of a number of structures and their $^{13}C$ NMR spectra in which each carbon atom is encoded and stored in a manner that correlates the chemical environment to the associated chemical shift. To predict a $^{13}C$ NMR chemical shift, the database is searched for the carbon atom which best matches the chemical environment of the carbon atom whose predicted chemical shift is sought. By repeating this procedure for each carbon atom in a structure, an entire $^{13}C$ NMR spectrum can be predicted.

The second approach is based on empirical modeling techniques. A linear model is computed of the form

$$S = b_0 + b_1X_1 + b_2X_2 + \cdots + b_nX_n$$

where $n + 1$ terms are summed to estimate $S$, the chemical shift of a specific carbon atom whose predicted chemical shift is desired. The $X_i$ terms are structural descriptors that encode some topological, electronic, or steric aspect of the chemical environment. The $b_i$ terms are computed by use of regression analysis techniques with a database of structures and spectra.

The database retrieval method is limited by the range of carbon atoms it contains. It does not have the capability of interpolation. The empirical modeling method can predict chemical shifts very precisely, but a given model is only applicable to a narrow range of chemical environments. To overcome these limitations, work in the authors' laboratory is currently directed to combining these two spectral prediction approaches in an automated manner.

This presentation focused on methodology for encoding the environment of each carbon atom in the database and developing automated techniques for selecting a subset of carbon atoms for use in building chemical shift models of the type described above. This capability is important, as current plans envision the calculation of a model for each carbon in the structure whose $^{13}C$ NMR spectrum is to be predicted. The environmental coding scheme currently under investigation uses a numerical vector-based approach. Each vector element is a calculated number which attempts to represent the chemical environment at a certain distance from the carbon atom being described. A vector is calculated and stored for each carbon atom in the database. Euclidean distance calculations can then be used to select atoms from the database based on the similarity of their environment vectors. This methodology was evaluated by use of a database consisting of 30000 structures and spectra.

### Sudden impact: the use of automated SFE for environmental applications

*Joseph M. Levy, Lori A. Dolata and Robert M. Ravey, Suprex Corporation, 125 William Pitt Way, Pittsburgh, PA 15238*

Supercritical fluid extraction (SFE) has proven its applicability as a sample preparation tool, especially with regards to environmental situations with the acceptance of SFE for use in Total Petroleum Hydrocarbons (TPHs) in soil extractions. Besides this achievement, other methods involving analytes, such as Polynuclear Atomic Hydrocarbons (PAHs). Pesticides and PCBs need to be compared to established EPA methods and to be evaluated for equivalency, accuracy and precision. The advantages of SFE (i.e. time savings, solvent cost savings) can be distinctly augmented by the development of true automation for greater sample throughput which can be especially important for the environmental market.

This paper presented data that demonstrated quantitative equivalency of SFE to conventional Freon-113 liquid solvent extraction for TPHs using non-toxic supercritical carbon dioxide and the proposed EPA method 3560. Statistical data was generated by Infrared (IR) and gas chromatographic analysis off-line after running multiple (44) samples using a newly designed automated SFE. Also, results were presented for the SFE method optimization that was achieved for dry and wet ($>30\%$ moisture) soil matrices.

Furthermore, SFE of other various environmental applications such as PAHs, Pesticides, and PCBs are priority candidates for future EPA method approval. SFE utilizing $CO_2$ is favourable due to its compatibility to many chromatographic detectors including flame ionization (FID), mass spectrometry (MS) and electron capture (ECD). For each application, using automated SFE with unattended operation, method optimization was achieved and statistical data was generated based quantitation that satisfied EPA QC acceptance criteria and equivalency of SFE to Soxhlet.

### On-line analysis method for the determination of cyanide in the presence of sulphide in wastewater using a novel amperometric flow-through detector

*Paul Karges, Eppendorf North America, Inc., 545 Science Drive, Madison, WI 53711*

The analysis of ppb levels of free cyanide in wastewater is most commonly performed by a colorimetric method as specified in the EPA procedure 335.2. Although this method has been automated, the time-consuming sample cleanup steps which include precipitation of sulphide and distillation to remove other impurities still must be performed manually.

An on-line flow injection analysis (FIA) method which injects raw wastewater samples directly (after on-line filtration) into the carrier stream has been performed using a membrane separator followed by amperometric detection. The FIA method which is capable of analysing from 50 to 1000 ppb of cyanide can be adjusted to other ranges by changing the size of the inject loop. Based on the signal-to-noise level, the detection limit appears to be around 10 ppb. The method consists of injection into a stream of 0.2 N sulphuric acid which contains 3 millimolar copper sulphate. The cyanide present in the sample is then separated from the sample matrix by passing through a teflon membrane into a continuously moving stream of 0.1 N sodium hydroxide. The sodium hydroxide carrier then carries the cyanide ion to the 3 electrode amperometric cell operating at $-52.15$ mV. Details of the membrane separator and amperometric cell were discussed.

### A universal approach for determination of physical and chemical properties of water by near-IR spectroscopy

*Jie Lin and Chris W. Brown, Department of Chemistry, University of Rhode Island, Kingston, RI 02881*

Determinations of physical and chemical properties of water and their functions of temperature have been traditional tasks in physical chemistry. One specific method usually can only measure one property at a time. Spectroscopic methods have not been explored for both manual measurement and remote monitoring of these properties.

Theoretically, all physical and chemical properties of water are determined by its structures and are reflected in the near-IR spectrum of water. It is expected that these properties can be determined by the near-IR spectra of water and, therefore, a universal spectroscopic approach can be developed.

Previously, the authors had developed a fibre-optic sensor and used it to measure the near-IR spectra of water from 5 to 85°C. In this study, those spectra were correlated with the physical and chemical properties of water. The 15 properties studied included density, refractive index, dielectric constant, viscosity, surface tension, vapor pressure, sound velocity, isothermal compressibility, thermal expansivity, thermal capacity, thermal conductivity, enthalpy, free energy, entropy, and ionization constant. The correlation methods used include principal component regression (PCR), partial least squares (PLS) and multilinear regression (MLR). Very good correlations were found between the near-IR predicted values of all these properties and those obtained by other methods. This study demonstrated that these 15 properties of water can all be determined simply by measuring a set of near-IR spectra of water. This indicates that near-IR spectroscopy can be used as a universal method for the measurements of physical and chemical properties of water in the laboratory or in industrial process control. Remote sensing can be accomplished by means of a fibre-optic sensor.

### Remote monitoring of physical and chemical properties of NaCl solutions with a near-IR fibre-optic sensor

*Jie Lin and Chris W. Brown, Department of Chemistry, University of Rhode Island, Kingston, RI 02881*

Monitoring physical and chemical properties of electrolyte aqueous solutions are very important in both laboratory investigations and industrial applications. Many methods are available for this purpose, but they usually can only determine one property at a time. Spectroscopic methods have not been explored for the remote monitoring of these properties, and a universal method is desired for monitoring several properties simultaneously.

The dissolution of electrolytes in water causes changes in the intermolecular interactions of water, and these changes will be reflected in the changes of the near-IR spectrum of water. Therefore, it is expected that the physical and chemical properties of aqueous solutions of electrolytes can be determined simultaneously from the near-IR spectra.

Previously, the authors had investigated the remote monitoring of NaCl in aqueous solutions using a near-IR fibre-optic sensor. In this study, those spectra were correlated with different properties of the NaCl solutions including density, refractive index, viscosity, specific electric conductance, freezing point depression, osmolality, activity coefficient, and water concentration. Very good correlations were found between the near-IR predicted values and those determined by other methods. The correlation methods applied include principal component regression (PCR), partial least squares (PLS) and multilinear regression (MLR). The results of this study demonstrated that several properties of NaCl solutions can be simultaneously determined with near-IR spectroscopy.

### Multivariate techniques for in-line near infrared analysis of polymer melts

*David A. Stewart, Atul Khettry, Jatender Batra and Marion G. Hansen, University of Tennessee, 419 Dougherty Engineering, Knoxville, TN 37996*

Peak absorbance changes correlated to changes in species concentration have been the norm in applied spectroscopy, while baseline shifts have been more of an inconvenience. Taking the first or second derivative of the spectra eliminates these baseline shifts. However, with multivariate techniques becoming more readily available, repeatable baseline changes may now be monitored and correlated to specific physical changes. An example is the addition of titanium dioxide ($TiO_2$), a white colourant for polyethylene terephthalate (PET), causing a baseline increase but very little change in relative peak absorbance in the near infrared (NIR) region between 1200 nm and 2200 nm. This baseline increase is attributed to the $TiO_2$ scattering light rather than having a specific wavelength absorbance in this spectral region. The resulting change, however, is significant enough to be correlated to the concentration of $TiO_2$ in the flowing polymer.

A specially modified flow cell, housing two fibre optic probes, was mounted downstream of a single screw extruder and gear pump. This arrangement allowed for in-line spectral measurement of flowing polymer. Mixtures of filled and unfilled PET, in increments of 10%, were run through the system. The concentration of filler was normalized between 0 and 100% with 100% corresponding to 'pure' filled PET.

Multivariate approaches involving the use of singular value decomposition (SVD) to perform principle component regression (PCR) and partial least squares regression (PLS) were used to quantitatively determine the $TiO_2$ content in the PET melt stream. A calibration model comprised of 51 samples with absorbance values at 350 wavelengths (1201–1550 nm) was developed. The predictive ability of the calibration model was tested against a validation set of 10 samples. The calculated standard of prediction (SEP) values for PCR-SVD and PLS-SVD were 0.9558 and 0.7462. The robustness of the calibration model can be judged from the plot as well as the SEP values. The results are satisfactory for both techniques with PLS-SVD being more accurate.

### Measurement of total sulphur contaminants in industrial gas streams

*John D. Ray, Neil Johanson and Richard S. Hutte, Sievers Instruments, Inc., 1930 Central Avenue, Boulder, CO 80301*

Sulphur compounds are often found as contaminants in industrial gas streams. For example, sulphur compounds in refinery gases can poison expensive catalysts or sulphur compounds in carbon dioxide can lead to off-flavours in carbonated beverages. The measurement of total sulphur can be used as a quality control technique when an upper limit specification can be given. A sulphur chemiluminescence detection method was used with an enhanced burner and a sampling system to determine total sulphur in some representative gas streams. Carbon dioxide samples from beer fermentation were tested for total sulphur and the results compared to total sulphur determined by gas chromatography. In both process streams and head space samples the total sulphur determined was comparable. Carbon dioxide was found to not interfere with the analysis.

Several different hydrocarbon streams were tested, including natural gas, propane, and ethylene. In each case the total sulphur method provided values equivalent to the gas chromatographic determined values. Introduction of excessive amounts of hydrocarbons into the burner were found to cool the flame and reduce sensitivity. By limiting sample introduction to less than 1% of the flame gases, interference could be eliminated.

### Determination of trace metals in solid samples via on-line digestion flame atomic absorption spectrometry

*Thomas J. Gluodenis, Jr. and Julian F. Tyson, Department of Chemistry, University of Massachusetts, Amherst, MA 01003*

A flow injection system incorporating a stopped-flow microwave-heated reactor has been constructed for the preparation of solutions for subsequent analysis by atomic spectrometry techniques. Slurry samples are injected into the manifold and transported into a glass reactor mounted inside a microwave oven. Nitric acid is flushed into the reactor which is then sealed and the contents heated at 400 psi for 5 minutes. During this period the pressure is continuously monitored. After cooling, the reactor is vented and the contents flushed out into a volumetric flask and diluted to volume. The procedure has been evaluated by the determination of some minor elements in cocoa powder (for which results were compared with two other digestion procedures), horse kidney (International Atomic Energy Agency H-8) and coal (NIST SRM number 1632b). A comparison of results showed no significant difference (95% confidence) for the trace element content of these materials between the flow injection method and two other methods of sample preparation. An analysis of variance shows no indication of sample or slurry inhomogeneity. Low recoveries were obtained for the coal material, perhaps due to incomplete dissolution of the silicate constituents.

### The analysis of environmentally regulated components of petrochemicals by near infrared spectroscopy

*Timothy M. Davidson, Larry McDermott and Rosemary Hake, The Perkin Elmer Corporation, Process Analytical Instruments, 2771 N. Garey Avenue, Pomona, CA 91767*

New US regulations have required gasoline to be blended with oxygenates and the levels of benzene in gasoline to be reduced. The addition of oxygenates to gasoline is expensive and requires tight control to keep production costs at a minimum. Therefore, on-line blending measurements of oxygenates and benzene are needed to reduce cost and to comply with enforced regulations.

The major types of oxygenates used in gasoline are methyl-*t*-butyl ether, *tert*-Amyl methyl ether, methanol and ethanol. Recent regulations require oxygen to be added to gasoline at a level of 1.8 to 2.7% in geographical regions with documented high carbon monoxide concentrations. Local and regional agencies, in such states as California, are imposing additional mandates. For example, by 1996 all gasoline sold in the state of California must contain on the average 0.8% benzene.

Diode array based near infrared process instrumentation is ideal for on-line quantitative analysis due to its ruggedness, analysis speed (less than 15 s), stability and simplicity. This presentation discussed analytical figures of merit of using the third overtone of the near infrared spectrum for the analysis of oxygenates and benzene in synthetic gasoline. The analytical performance was discussed in terms of detection limits, linearity and stability.

### In-process particle size distribution measurements using laser/optical techniques

*Donald J. Holve, Thomas L. Harvill and Michel P. Bonin, Insitec, 2110 Omega Road, Ste D, San Ramon, CA 94583*

In-process measurement of particle size and concentration distributions provides continuous analysis and quality control of a product stream and can be used to monitor particulate emissions. As process production rates continue to improve, the delay between laboratory analysis and process correction of the product stream becomes more significant and costly in many applications. Elimination of sample handling and operator manipulation is now possible for most pneumatic flows using optical methods which are properly interfaced with the process stream. Insitec has developed a range of laser-optical instruments for application in difficult environments. Two specific instruments and recent measurement results are discussed.

A single particle counting instrument known as PCSV-P (Particle Concentration, Size, Velocity-Probe) was modified to obtain size distribution and concentration measurements in the exhaust of a coal-fired Magneto-HydroDynamic (MHD) test facility at the University of Tennessee Space Institute. The MHD process operates at high temperatures (2700 K), and thus produces a very fine vaporization-condensation flyash aerosol (less than 3–5 microns top size). Detailed size distribution measurements are required to better understand the performance of the emissions control systems. Real time PCSV-P measurements have shown quantitative agreement with infrequent mass sampling results. Recent work extends the PCSV-P size and concentration range to particle sizes below 0.2 microns at number densities exceeding $10^8$/cc. Measurements of detailed size distributions upstream and downstream of the emission control systems provides particle removal efficiencies as a function of particle size.

A second instrument known as EPCS-F (Ensemble Particle Concentration & Size-Flange) has been used to obtain detailed size distribution measurements in powder production facilities at one second intervals, suitable for automatic feedback control. This fast data acquisition rate and display allows for real-time particle classification control on any user-selected element of the size distribution. Recent results for air-bag propellents, toners, resins, and pharmaceuticals were discussed.

### Comparison of on-line particle size analysis to laboratory results

*Mark R. Bumiller, Malvern Instruments, Inc., 10 Southville Rd., Southboro, MA 01772*

Particle size analysis of various samples was performed both in the laboratory and on-line using laser diffraction. The principle of the laser diffraction method was reviewed, with attention paid to the effect of refractive index on final results.

Data was presented from field analysis in industrial processes, from a test stand designed for on-line analysis, and from laboratory experiments. The results from the laboratory and on-line analysis were compared and discussed. Results were also compared to other methods, including sieve analysis. Sampling and suspension methods were considered, as were criteria for choosing whether to analyse powders dry or suspended in liquids. Proper orientation and dispersion of dry powders were investigated with results shown from both proper and improper techniques.

Materials investigated included taconite (iron ore), powdered garnet, and plastic resins.

### On-line determination of hydrofluoric acid: chemical fuse versus near-IR approach

*Hernán Prieto, Adriano Parisi and Marcelo Carrillo, Departamento de Análisis y Evaluación, INTEVEP S.A., Apartado Postal 76343, Caracas 1070A, Venezuela*

Hydrofluoric acid is used in the petroleum industry as a catalyst in alkylation plants. This process converts light alkenes such as propylene and butylene, as well as linear paraffins into heavier and branched paraffins such as isooctane and isoheptane. During operation, the acid concentration has to be maintained at a level between 90–92% to assure optimal catalyst activity and reduce corrosive effects. For such purposes, not only the acid strength has to be measured but also its water content needs to be assessed. Both analytical parameters are usually monitored off-line by acid-base and Karl-Fisher titration. Those procedures are slow and hazardous, mainly due to the sampling step and the precautions that need to be taken in handling the sample during the analysis.

In this paper two alternative analytical methods to measure HF concentration were devised. In both cases it was required that the methods could be easily transferred to an on-line type of analysis. In the first one, an optical sensor based on the SPANDS reaction, was constructed. A piece of plastic optical fibre was bent in 'U' shape and embedded in epoxy. One branch of the fibre was grooved to get an absorption cell path of about 5 mm in length. The cell was surrounded by a small bag made of latex coming from piece of a laboratory protective glove and it was filled with a solution of the SPANDS reagent. The device was immersed in the aqueous solution and the leaching of the dye was monitored in the visible range. On a second approach, a tygon tubing was pressed between two metallic plates coupled to SMA connectors from a 500 μm core diameter glass fibre. The sample was pumped through the tubing and its near infrared spectrum was taken. The variation in the spectrum was related to the concentration of HF and water via multivariate calibration in the Partial Least Square (PLS) mode.

In the first approach the optrode is sensitive to sudden changes in an otherwise stable hydrofluoric acid stream but has a finite life given by the amount of SPANDS contained in the membrane. This drawback was overcome by supplying fresh reagent to the sensor through a small diameter tubing using a peristaltic pump. However, this system works only for low concentration of fluoride. In the second method a straight spectroscopic measurement is performed and there is no consumption of reagent. In this case the calibration was performed up to 48% HF in aqueous solutions.

The preliminary laboratory results suggest that the near infrared method has good possibilities to be transferred on-line for process control and monitoring. However, with a very modest investment, the SPANDS based sensor could potentially be used as a chemical fuse to detect anomalous HF concentration in waste streams.

### On-line column preconcentration of rare earth elements for ICP-AES simultaneous determination

*N. M. Kuz'min, V. M. Pukhovskaya, O. N. Grebneva, Vernadsky Institute of Geochemistry and Analytical Chemistry, Kosygin str. 19, Moscow, 117975 GSP-1, Russia; G. I. Tsysin and Yu. A. Zolotov, Moscow State University, Chemistry Department, Lenin Hills, Moscow, 119899, Russia*

Sorbents containing conformationally flexible aminocarboxylic functional groups were used to develop an efficient on-line preconcentration system for ICP-AES determination of rare earth elements. The influence of the chelating centre structure and sorbent matrix on the concentration efficiency was investigated. Recovery of 14 REE as a function of pH, salinity and flow rate for a synthetic mixture containing some matrix elements was examined. Dynamic capacities were evaluated. The time of analytical response is not more than 40 s.

To enhance the selectivity of REE determination, for example in geological samples, sulphosalicilic acid was used as a complexing agent. In its presence, 100% recovery of REE from high-salinity solutions was achieved. The recovery of other elements (Fe, Ti, Al, Zr, etc.) was significantly reduced.

Commercial software was applied to create a program of on-line simultaneous ICP-AES determination with RSD of 3–5% at levels of 10–11 ppb respectively.

### The NIST consortium on automated analytical laboratory systems—standards for automated systems used in analytical chemistry

*Gary W. Kramer, National Institute of Standards and Technology, Chemistry Building 222, M/S A-343, Gaithersburg, MD 20899*

The National Institute of Standards and Technology has joined with interested parties from the private sector and other government agencies to form the Consortium on Automated Analytical Laboratory Systems. CAALS overall goal is to foster the development of automation in analytical chemistry.

As one of its initial objectives, CAALS has undertaken, with guidance from its members and others in the analytical instrumentation community, the task of identifying, defining, and promoting general guidelines and standards in critical areas of samples, data, and control information interchange for analytical instruments. From the CAALS viewpoint, fully automated analytical systems (those which take in raw samples and return chemical information) may be constructed most appropriately by interconnecting modular instruments which have been designed specifically as system—instead of user-operated, stand-alone—devices. CAALS is determining the requirements and specifications for interfaces between such analytical modules and their controllers.

CAALS is developing the requirements and specifications for both the modules (known as Standard Laboratory Modules or SLMs) and their the workcell controllers (called Test Station Controllers or TSCs). Current developments include a protocol and syntax for communicating between a TSC and its SLMs and a list of requirements for instrument controllability. 'CAALS-Ready' instruments that meet these requirements will be easy to incorporate into today's systems and can be upgraded into the fully compliant CAALS modules of tomorrow. This presentation described progress to date.

### NIST CAALS: description of the standard interface within an automated chemistry workcell

*F. R. Guenther and M. L. Salit, National Institute of Standards and Technology, Chemical Science and Technology Laboratory, Chemistry B158, Gaithersburg, MD 20899*

Over the past three years the National Institute of Standards and Technology (NIST) has hosted the Consortium on Automated Analytical Laboratory Systems (CAALS). The CAALS working group on modularity standards has developed preliminary specifications for a standard interface between a controlling entity and laboratory equipment to facilitate the development of automated systems. This standard describes the behaviors, command semantics, command syntax, and communications protocols for both the supervisory process and the subsystem being controlled. Adherence to a standard which encompasses these domains will allow a developer of automated systems to integrate components of the system using standard re-usable tools while a minimizing the special attention to each device.

The recommended behaviours characterize the responsibilities for 'good citizenship' in an automated system for both the apparatus being controlled and the controller. Command semantics describe the messages which flow between the entities. The command syntax is the format of the messages, and the communications protocol specifications provide a single Applications Programming Interface (API) and recommended practices for implementing communications links between the controller and the apparatus.

This paper described the NIST implementation of the standard interface. The implementation created two software object libraries in C++ for the Microsoft Windows operating environment. The first library provides a standard communications software interface between the controlling process and the controlled apparatus. The current communications links supported by this library are EIA232 and Windows Dynamic Cata Exchange (DDE). The second library provides the CAALS standard behaviour, semantics, and syntax required of a modular instrument. This library is responsible for presenting the CAALS interface to the controlling process. Using these two libraries it is possible to convert existing instruments ito CAALS modules by providing a software layer between the two libraries and the existing instrument interface. This software layer, the 'Glue' layer, translates command requests from the CAALS library into native device commands. Along with a description of the two software libraries, several examples of these 'modularized' instruments were described.

### NIST CAALS: controlling automated chemistry workcells

*M. L. Salit and F. R. Guenther, National Institute of Standards and Technology, Chemical Science and Technology Laboratory, Chemistry B222, Gaithersburg, MD 20899*

An approach for controlling automated chemistry workcell has been developed which borrows from process control and automated manufacturing control strategies. The scope of this approach extends from a description of the chemical methods to a set of tasks for an automated workcell controller to co-ordinate through the execution of these tasks by the subsystems of the workcell.

The problem can be described as having three different conceptual strata—method description, method scheduling and method execution. The interactions between and behaviours of the method description and the method execution levels are being described by CAALS specified interfaces. However, method scheduling is merely a requirement of performing the method, and as an implementation based requirement will not be captured in a specification. Example implementations will serve to describe approaches which effect the required behaviours.

A potential method description language is based upon a directed graph approach which is similar to a PERT chart. This graphical description must be decomposed into a list of tasks, which then must be executed according to the logical scheme embodied in the graph. The logic must include the fundamental serial precedence relationship (for example one must obtain a sample before aliquoting from it), condition predicates (for example one must wait for a GC oven to reach its start temperature before injecting a sample), branching constructs for parallel tasks (for example centrifuge one aliquot *and* filter another) and conditional tests (for example use either the centrifuged *or* the filtered aliquot).

The requirement of a directed graph language and the implementation of a controller to interpret, schedule and execute these graphs was discussed.

### LIMS standards: the ASTM LIMS guide

*James B. Powers, Jr., Chairman ASTM E31.40 LIMS Guide Subcommittee, Associate Director QA Computer Applications, Wyeth-Ayerst Laboratories, Inc., P.O. Box 597, Paoli, PA 19301*

The *ASTM LIMS Guide* is the first industry standard developed specifically for the Laboratory Information Management System discipline. This paper described the content of the LIMS Guide and and how the document can be used.

The purpose of the *ASTM LIMS Guide* includes: (1) help educate new users of Laboratory Information Management Systems (LIMS), (2) provide standard terminology that can be used by LIMS vendors and end users, (3) establish minimum requirements for primary LIMS functions, (4) guidance for the specification, evaluation, cost justification, implementation, training, and documentation, (5) provide an example of an analytical data/information model and (6) provide a LIMS functional check list suitable for a Request For a Proposal (RFP).

Information contained in the Guide will benefit a broad audience of people who work or interact with a laboratory. New LIMS users can use this guide to understand the purpose and functions of LIMS. The guide can help prospective LIMS users in understanding terminology, configurations, features, design and costs. Individuals who are purchasing a LIMS can use this guide to identify function that are recommended for specific laboratory environments. LIMS vendor R&D staffs can use the guide as a tool to evaluate, identify and correct areas that need improvement. LIMS vendor sales staffs can use the guide to accurately represent functions of their LIMS product to prospective customers.

### Proposed ASTM standards for computerized chemical structural information

*Charles E. Gragg, Regulatory and Scientific Information Department, Burroughs Wellcome Co., Research Triangle Park, NC 27709-4498*

Within ASTM (American Society for Testing and Materials) committee E49 on Computerization of Material and Chemical Property Data, subcommittee E49.51 on Chemical Structural Information (CSI) has drafted a new standard specification for computerized CSI, with focus on information content.

The significance and use of the proposed standard specification include exchange of CSI among computerized Analytical methods, Publications, Databases, Molecular Modeling, Structure Activity Relationships (SAR) and Structure Property Relationships (SPR).

Inclusion of appropriate amounts and types of useful chemical structural information in files or datasets is the goal of the proposed standard.

Concepts involving complex chemical structures such as mixtures, polymers, reactions, patents and 3D models were presented to illustrate the provisions of the latest draft.

### The future of usability engineering and standards in lab automation

*Rich Lysakowski, Digital Equipment Corporation, Four Results Way, MR04-2/C17, Marlboro, MA 01752*

'Open systems' and 'ease of use' are buzzword phrases that have been kicked around for many years, yet why have there been only incremental improvements in openness the past few years, and why is the laboratory a hodge-podge of different interfaces that together hinder easy-of-use?

Is it because chemists have not made their needs clear to vendors? Probably not. Is it because the end users' needs do not address vendors' needs to make money? Probably not. Is it because devices that chemists use are too complicated or too young in their lifecycles to standardize? Probably not. Are these devices too vendor-specific to be fully integrated into open laboratory systems? No, because are some good examples of truly open and integratable laboratory instrument components. Why are not there more examples of easily integrated pieces and how can we change this paradigm of closedness and complexity?

Part of the problem is that there is much confusion in the scientific community about what 'open systems' means. Some people think an 'open' computer system equates to Unix or DOS even, though they are proprietary operating systems. The confusion about open systems is unnecessary when one loosk at the characteristics of open systems.

Another part of the problem is that concepts of usability engineering are understood by too few companies in the instrument industry. Some think that usability is achieved by designing a piece of equipment that is easy-to-use, without looking at that piece of equipment in the context of the entire workplace.

What are the characteristics of open systems and usability engineering that are missing from the marketplace? How can end users and vendors partner more effectively to develop laboratory equipment and software that are much easier to use, less time consuming, and truly 'open'? We know that scientific productivity is beleagured by the lack of consistency in instrument interfaces. However, what are the proper roles of consistency and information hiding in software interfaces and front panels? This productivity gap must be filled over the new few years. The place to start is education.

This paper covered the fundamental of Usability Engineering (ease-of-use) and characteristics of Open Systems not being addressed by vendors yet. Points were made about productivity improvements that can be achieved by careful application of usability engineering and standardization. A practical picture that works for the future of open laboratory systems (and their support) was also presented.

### Determination of elements in the far UV by AAS using a pulsed continuum source and a linear photodiode array

*Clare M. M. Smith and James M. Harnly, USDA, Nutrient Composition Laboratory, Bldg. 161, BARC-East, Beltsville, MD 20705*

A system for continuum source AAS incorporating linear photodiode array (LPDA) detection has been developed which offers analytical performance comparable to that of line source AAS. Using this system, wavelength integrated absorbance, A, was calculated for each scan of the array. Time integration was achieved by summing A over the atomization cycle. The higher quantum efficiency and multiplex advantage obtained using the LPDA detector allows improved SNR to be obtained in the far UV. Detection limits of the same order as line source AAS have been obtained and characteristic mass values achieved offer an advantage over line source AAS at very short wavelengths. The useful analytical range of calibration graphs is extended with LPDA detection compared to PMT detection due to the 2-dimensional integration of the absorbance signal. Further characterization of the system was reported. This included discussion of the relative merits of operating the xenon arc lamp in continuous and pulsed modes and applications of the calibraph graph capabilities.

### Factors affecting sample throughput for an ICP-OES system with a segmented-array CCD detector

*Kenneth J. Fredeen and Cindy Anderau, The Perkin-Elmer Corporation, 761 Main Avenue, Norwalk, CT 06859-0215*

Factors affecting analytical sample throughput have been investigated for the Optima 3000™ inductively coupled plasma (ICP) optical emission spectrometer. This system includes a computer-controlled ICP source, an echelle-based spectrometer, and a custom-made two-dimensional array detector called the Segmented-array, Charge-coupled-device Detector (SCD). Each of these system components has been designed and optimized specifically for ICP-OES applications.

The Optima 3000 optical system and SCD provides access to over 5000 atomic emission lines, including over 200 prominent ICP emission lines. Up to 224 designated spectral regions, reach of which covers analyte emission lines plus nearby background emission, can be measured simultaneously by the system. One obvious advantage of this type of design is that a great amount of background-corrected spectral data can be collected in a relatively short period of time.

Besides the rapid spectral data collection speed, other features specific to this system can have a significant impact on overall sample throughput. Measurement noise is reduced, compared to other solid-state detectors used for ICP-OES, by using inherently 'quiet' CCD/CMOS technology and by placing signal amplifying and processing electronics on the face of the SCD. This low-noise characteristic of the SCD allows the analyst to use shorter integration times to achieve low detection limits and good measurement precision. The system also incorporates an optional autointegration algorithm that determines optimum integration times for each sample. This allows samples with high analyte concentrations to be measured more rapidly, while good detection limits are retained for samples with low concentrations.

The Optima 3000 software includes among its many advanced features a multivariate spectral interpretation routine called Multicomponent Spectral Fitting (MSF). The use of MSF can improve sample throughput by correcting for spectral overlaps without the inaccuracies associated with traditional interelement correction (IEC). MSF can also be used in certain cases to improve detection limits and measurement precision.

Factors that affect overall data collection speed and figures of merit such as detection limits, precision, accuracy, and dynamic range, were studied. These factors included sampling time, interference correction methods, and sample introduction parameters. By understanding the effects of these factors, it was possible to maximize the sample throughput for the applications studied while meeting the required data quality objectives.

### Analytical advantages of coupling simultaneous atomic emission spectra from the inductively coupled plasma with multivariate data processing

*Juan C. Ivaldi and Thomas W. Barnard, The Perkin-Elmer Corporation, 761 Main Avenue, Norwalk, CT 06859-0293*

Multivariate data processing methods have been used to interpret the spectra from Inductively Coupled Plasma (ICP) atomic emission spectrometers. The benefits of multivariate data processing, compared to peak height or single bandpass determinations, have been previously discussed in connection with sequential instruments primarily because scanned spectra are available at the output of the system. Prior work with spectral simulations shows that improvements in precision and detection limits are expected as a result of the use of all of the spectral information available within the spectral window. Spectral simulations were also used to predict that further improvements in the quality of the data can be achieved by using a detection system that records simultaneous emission spectra.

In this work, an ICP instrument equipped with an echelle spectrometer and a custom segmented array CCD detector provides simultaneous spectral data which are well-suited to the appoication of multivariate data processing. Least-squares digital filtering was used to reduce the data. With this system, the spectral background is recorded simultaneously with the analyte emission signal. By coupling the simultaneous data collection with a multivariate data treatment, significant improvements in analytical performance are possible through the reduction of correlated flicker noise originating in the ICP source. The results of experiments conducted with this ICP system were presented.

### Flow injection analysis of calcium in microdialysis samples

*J. S. Hall and J. B. Justice, Jr., Emory University, Department of Chemistry, 1515 Pierce Drive, Atlanta, GA 30322*

Microdialysis has emerged as a widely used method for *in vivo* sampling. In quantitative microdialysis, the point of no net flux method appears to be very useful for determining basal levels of substances and assessing their dynamics in the extracellular space. The analytical method employed for determining calcium concentrations in this experiment is flow injection analysis with UV detection. The increase in absorbance is measured at 650 nm upon binding of calcium to the dye Arsenazo III. It has not established that interference from ions other than calcium is not a potential interference in this approach. First order regressions provide slope and intercept values subsequently used to solve for the point of not net flux (zero intercept on the y-axis). This *in vitro* application is shown to estimate a 1.2 mM calcium standard to be 1.22 +/− .02 mM (mean +/− SEM, n = 5). The slope of each curve is a measure of the *in vitro* efficiency or recovery at a given perfusion flow rate. The future direction of this work is the application of this methodology *in vivo* in order to obtain information about calcium dynamics in the brain.

### An automated sample mixing and delivery system for light scattering particle size analysis

*A. H. Clark, M. E. Gerard, R. W. Brill and J. G. Hoffman, Leeds & Northrup Co., A Unit of General Signal, North Wales, PA 19454*

In particle size analysis by optical means, it is necessary to prepare a slurry of particles and fluid with constant mixing and circulation through an analyser cell, which provides a uniform homogeneous suspension.

It is well known that a mixing tank can be utilized with a stirring impeller inserted in the tank to mix the particulate in a fluid. However, as the mixture is circulated by a pumping action from the tank to an analyser cell, non-uniformity of distribution and particle settling can occur.

The Automated Recirculator provides a fully automatic mixing and recirculating system with features that mix and recirculate without distortion of the particle size distribution. The device uses a non-impeller return flow mixer in conjunction with a sealess pump. The device provides (1) a unique method of producing uniform mixing and delivery of particles in a slurry, and (2) fast and complete dispersion from an in-stream ultrasonic probe. Various sizes, densities and viscosities are correlated by a $pD^2$ (density × diameter$^2$) factor to show the limits of uniform mixing. The in-line probe dispersion capability is compared to that of an ultrasonic bath.

The hydrodynamic flow distribution through the sample cell area was determined to show the criticality of the transition length in the cell structure. The effect shows how an incorrect design can considerably distort the distribution. The recirculator is a PC driven automated system for fill, dilute, clean, drain, deaerate, ultrasonic and flow for continuous operation. The recirculator/analyser system together can be pre-programmed to provide up to 100 different automatic analysis conditions with one-button operation.

Also, a brief description was included of how this non-hazardous recirculator accommodates Class D flammable fluids under the NFPA Code.

### On-line FTIR spectroscopic comparison of primary and secondary antioxidant levels in molten polyolefins via a high pressure, high temperature flow cell

*Robert A. Fidler, Flow Vision, Inc., 1911G Associates Lane, Charlotte, NC 28217*

Polyolefins have proven to be particularly susceptible to oxidative degradation. For this reason, antioxidants are commonly added to inhibit the oxidative process thereby increasing the useful life of many polymer products.

Analyses for the proper levels of antioxidants in polyolefins are commonly performed via traditional laboratory testing techniques. Drawbacks to such testing include long lag times for results and errors introduced in sample preparation.

However, FTIR analysis performed in a continuous mode via a high pressure, high temperature flow cell and polymer sampling system applied directly to the extruder or reactor opens up a real-time window on the additive compounding process. In addition, on-line examination of additive stability becomes possible.

This work demonstrated recent applications of on-line FTIR technology to the analysis of both primary and secondary antioxidant levels in molten polyolefins. Such on-line measurement offers real-time control capability of the compounding process thereby enhancing polymer uniformity and quality.

### The use of remote sensing FT-IR spectrometry to inspect the surfaces of ordinary materials

*G. L. Powell, Oak Ridge Y-12 Plant[a], Martin Marietta Energy Systems, Inc., Oak Ridge, TE 37831-8096*

Diffuse-reflectance, internal-reflectance, and external-reflectance FT-IR spectroscopies are powerful techniques for materials characterization and surface analysis provided the spectrometer can address the appropriate location on a specimen under conditions for which the resulting measurements is meaningful. The Spectropus™ system of remote sampling terminals for FT-IR spectrometers has been developed to obtain spectra from the surfaces of large flat or convex objects in ambient air or in environmental chambers with sufficient ease that meaningful statistical comparisons of spectra obtained from many locations on an object or from many objects can be made. This paper described recent additions to the Spectropus™ system, including an internal reflectance sampling terminal (TurboATR™), an automated external remote bench configuration (Quadrapus™) supporting four sampling terminals (two diffuse reflectance barrel ellipsoids, a 15° specular reflectance terminal with polarizer, and a 45° single reflection internal reflectance terminal with polarizer) and two ports for gas cells or future expansion.

Also described was a portable surface inspection instrument (Inspector®) consisting of a barrel ellipsoid diffuse reflectance sampling terminal attached to a MIDAC Illuminator® FT-IR spectrometer configured for 12-VDC operation and minimum size (299 mm by 200 mm by 600 mm) and weight (13 kg). The application of these techniques to practical inspection processes on ordinary materials was demonstrated using spectra obtained from paper, composite materials, sheet-metal, machined surfaces, and tubing. An evaluation of these spectra with respect to detecting surface contamination, evaluating films and near-surface regions of materials, sampling time, and the relative merits of internal-, diffuse-, and specular reflectance for particular applications were presented. The practicality of the technique for routine inspection of materials in a manufacturing environment was discussed.

### An automated GC/MS system for the analysis of volatile and semi-volatile organic compounds in water

*Allen K. Vickers and Lowell M. Wright, OI Analytical, PO Box 2980, College Station, TX 77841-2980*

The number of gas chromatographs/mass spectrometers used in environmental analyses continues to increase rapidly. This is due to the fact that gas chromatography/mass spectrometry (GC/MS) analysis is considered a definitive qualitative process, alleviating the need for further confirmatory analyses. Also, improved technology has resulted in affordable MS systems that are easy to operate. The increase use of GC/MS is especially evident in the analysis of volatile and semi-volatile organic compounds. These two types of methodologies (USEPA Methods 624 and 625) represent the bulk of environmental analyses.

One limitation of these GC/MS methods is that the instruments assembled to perform either volatile or semi-volatile organic compound analyses are not readily convertible from one method configuration to another. This can severely limit the profitability of the GC/MS system. In this paper, an automated Purge-and-Trap/GC/MS system was presented capable of performing both volatile and semi-volatile organic compounds analysis. Following USEPA guidelines, the analyser system was optimized through the use of a new injector system for the GC, the use of a capillary column, and computer/instrument interfacing.

### Coupling sample preparation to gas chromatographic analysis

*Philip L. Wylie, Charles R. Knipe, Patricia Castelli and Dennis Gere, Hewlett-Packard Co., 2850 Centerville Rd., Wilmington, DE 19808*

Supercritical fluid extraction (SFE) is gaining acceptance as an alternative to conventional extraction methods because of its speed, flexibility, and minimal use of organic solvents. The United States Environmental Protection Agency (USEPA) has recently proposed an SFE method for the extraction of total petroleum hydrocarbons (TPH) from soil, sludge, and sediment (Method 3560). This new method replaces a soxhlet extraction procedure which consumes large amounts of Freon.

Using the Hewlett-Packard 7680T Supercritical Fluid Extractor, up to eight soil, sludge, or sediment samples can be processed automatically. Extracts are delivered by the supercritical CO2 to a trap packed with silica. The trap is first rinsed by a non-polar solvent into a standard 2 mL autosampler vial. A second rinse by a more polar solvent removes the polar fraction and regenerates the trap. Gas chromatographic analysis is done off line, but the entire process from extraction through gas chromatography can now be coupled for full automation.

New software couples the HP 7673 Automatic Sampler tray and GC to the HP 7680T Automatic Sampler tray and GC to the HP 7680T Supercritical Fluid Extractor. To start an extraction/analysis sequence, capped empty vials are moved from the autosampler tray to the SFE instrument by the tray's robotic arm. The SFE extracts the first sample and rinses the extract into the waiting vials which are then returned to the GC autosampler tray. Using the autosampler's syringe, internal standard is added prior to GC analysis. The next sample is extracted while the first is being analysed by the GC. This automated process continues until all eight samples are extracted and analysed.

The coupling of off-line SFE to GC avoids problems associated with on-line SFE/GC such as overloading and the need to use very small well-characterized samples. Hewlett-Packard's off-line coupling of SFE to GC takes advantage of robotics and communications linkages built into the individual components to provide a total solution for the analysis of TPH in soil, sludge, or sediment. For example, up to eight samples can be analysed overnight with the complete analytical results available in the morning. If desired, a photoionization detector can be placed in series with a flame ionization detector so that a single analysis will provide BTEX (benzene, toluene, ethylbenzene, and xylenes) data in addition to a TPH value.

### A dedicated purge and trap/GC system for residual solvent analysis of pharmaceutical samples

*John W. Washall, Marge J. Matheson and Thomas P. Wampler, CDS Analytical, Inc., 7000 Limestone Rd., P.O. Box 277, Oxford, PA 19363-0277.*

Dynamic headspace, or purge and trap, techniques offer much to the analysis of residual solvents in manufactured goods. By purging the organics of interest from the sample, and concentrating them onto the surface of a trap, the use of a solvent for the extraction is eliminated, simplifying the chromatography and providing greater sensitivity. In addition, only the volatile organics are transferred to the gas chromatograph, leaving the sample matrix behind. In this way the technique serves as an automated sample preparation method in addition to providing enhanced sensitivity.

Liquid samples, or materials soluble in water, may be purged in the liquid state in a vessel designed to bring more purge flow below the surface, either through a ceramic frit or a glass impinger. The liquid may be purged at room temperature or heated to increase volatility of the analytes. Solid materials, and samples not soluble in water may be purged in the solid state. A weighed amount of the solid is placed into a glass tube which is then warmed while it is purged with carrier gas. In either case, the purge flow carries the volatilized organics to a sorbent filled trap, where they are adsorbed and then subsequently desorbed to the gas chromatograph. This desorption step is achieved thermally so that no additional solvents are added to the analysis. An internal standard may be added to either solid or liquid samples before purging, and it is then collected and desorbed along with the analytes.

All work described was performed using a CDS Model 600 combination headspace sampler and gas chromatograph. The samples—solids or liquids—were placed into the instrument which automatically collected the organic volatiles and analysed them by megabore capillary gas chromatography with a FID. Sample temperature and purge parameters were discussed, including the effect these have on the recovery of various common solvents.

### Automated SPE methods development

*Rolf B. Schlake and Al Kaziunas, Applied Separations, Inc., Allentown, PA 18103*

Solid Phase Extraction (SPE) has become the sample preparation technique of choice for efficient, reproducible operations. Even though the body of knowledge is increasing through the publication of more and more methods, new method development remains mostly a tedious operation. A matrix approach to methods development may provide a systematic, traceable approach to an optimal procedure.

The matrix approach suggested uses a different parameter in each column of the matrix and a different parameter in each file. With reverse phase SPE as a model, a series of techniques, using automated SPE software, will show how to reduce the time to arrive at an optimum procedure. C18, C8, C4, C2, C1 and Phenyl SPE cartridges are simultaneously processed by five different solutions. Data, showing which solutions provide optimum recoveries will be reported and discussed.

Similar configurations testing a variety of flow variations through SPE cartridges have been established. Data showing optimum flow rates for specific model procedures will allow the researcher to quickly determine the optimum technique.

Additional examples and set-up schemes were shown for normal phase and ion exchange mechanisms.

### Automated large volume solid phase extraction for environmental water samples

*David S. Williams and John Helfrich, Zymark Corporation, Zymark Center, Hopkinton, MA 01748*

Traditionally, water samples being analysed for organic pollutants were extracted by liquid/liquid techniques. Most liquid/liquid extraction methods used today were developed during the 1950s. These methods are designed using a separatory funnel to perform the extraction. Some years later, automation was applied to liquid/liquid extraction in the form of a continuous liquid/liquid extractor. Both methods are labour intensive and involve using large quantities of extraction solvent.

During the late 1970s laboratories started experimenting with liquid/solid extraction techniques. This technique, also known as Solid Phase Extraction (SPE), provides the same efficiency of extraction as liquid/liquid extraction, but with a 10 fold reduction in the amount of solvent consumed. The equipment used to perform SPE is a vacuum manifold. The advantage of reduced solvent usage is of great benefit to laboratories and the ozone layer, but the vacuum manifold method is still technique sensitive and has a limited capacity for samples containing suspended solids.

A new device has been developed for SPE that automatically conditions the SPE cartridge, loads the sample onto the SPE cartridge, and elutes the SPE cartridge without operator intervention. Samples are delivered to the SPE cartridges by positive pressure pumps allowing higher loading pressures than a vacuum system. By incorporating a microprocessor into the unit, precise sample load rates can be attained. This ensures that the interaction between sample and stationary phase is kept constant. This consistency helps maximize analyte recoveries and precision.

This paper discussed the operation of the device and provide recovery data from water samples.

### SPE methods development: different approaches for automated verses manual systems

*Al Kaziunas, Applied Separations, Inc., Allentown, PA 18103*

Solid Phase Extraction (SPE) sample processing is accomplished by sequentially passing conditioning, wash, and elution solvents through prepackaged SPE cartridges using a vacuum manifold. The solutions are manually pipetted into the SPE cartridges and simultaneously aspirated through the sorbent bed using vacuum as the motive force. In addition, many methods require air drying of the sorbent packing between the aspiration of the immiscible solvents.

The manual processing of SPE samples on a vacuum manifold is a time-efficient batch technique, but variable flow rates, solvent volumes, and air drying can lead to unreproducible recoveries.

Automated processing of SPE samples can achieve reproducible results by controlling flow rates and drying times, but most robotic systems process samples one at a time in an inefficient manner.

Factors effecting reproducibility and throughput were compared between SPE methods developed on manual processing systems and automated systems including the isolation of pesticides from water and 9 COOH THC from urine. Data concerning reproducibility and throughput were evaluated and a rationale for automated methods development was discussed.

### Automated drug dissolution monitor employing multiple fibre optic sensors and a UV/VIS diode array spectrometer

*Chi-Shi Chen and Chris W. Brown, Department of Chemistry, University of Rhode Island, Kingston, RI 02881*

Dissolution testing is used to assess the rate of release of active ingredients with respect to time. It is one of the most widely performed analysis in pharmaceutical laboratories. Previously, we have shown that the drug dissolution testing can be directly monitored using UV spectra. Aliquots from dissolution vessels are automatically transferred to a UV-visible diode array spectrometer, spectra measured and the aliquots returned to the testing vessels. A full spectrum calibration method based on principal component regression is used to simultaneously determine the concentrations of active ingredients and to account for interferences due to excipients in a tablet formulation.

Previously, the authors demonstrated that the traditional pumping system could be replaced with a fiber optic interface between the spectrometer and the sample. In that study, a single sample vessel was used. The authors are currently using a testing system with six vessels connected to a Beckman Model DU-7500 diode array spectrometer via six fiber optics. A bifurcated fiber optic bundle is used to transfer the light from the source to the dissolution vessels and is multiplexed so that spectra of each sample can be measured periodically. The multiplexing is preformed by mounting each of the six fibers into the cell holders of a standard Auto 6-sampler in the Beckman Model DU-7500. Software was written to automatically control the multiplexer, the data acquisition and the data processing. Results from this new fiber optic interface system on two commercial formulations were compared with those obtained previously with the traditional pumping system.

### A new approach to automated peak purity analysis in HPLC using diode-array detection

*Anton C. J. H. Drouen, Hewlett-Packard GmbH, Hewlett-Packard Strasse, W-7517 Waldbronn 2, Germany and Hans-Jürgen P. Sievert, Hewlett-Packard Company, Little Falls, PA*

Traditionally, the information content of ultraviolet (UV) spectra has been viewed as very limited, especially where spectral features are quite similar, for example, with different members of a class of compounds, or where impurity levels or signal-to-noise ratios are low.

Based on some theoretical considerations a purity check which uses superficially similar UV spectra; without the need for biased, operator-dependant selection of parameters has been developed. Theoretical and practical examples will be given of successful analysis at impurity levels lower than 1%.

### The evaluation of assay ruggedness using a factorial design approach

*Anne M. Warner, Eli Lilly and Company, Analytical Development, P.O. Box 685, Lafayette, IN 47902*

Assay ruggedness refers to the reproducibility of results obtained when using a particular assay method that is subject to operational variables. There are a number of continuous variables that may affect the ruggedness of chromatographic methods. A statistical approach to ruggedness evaluation affords an efficient way to determine: (1) which factor(s) have a significant influence on the assay results, and (2) how the factor(s) can be controlled to meet the requirements of the method. This statistical approach has been used to evaluate the intralaboratory ruggedness of the high performance liquid chromatographic assay for related substances in a pharmaceutical compound under development. A mathematical model was generated to predict how factors found to be significant affect the results. A useful parameter for system suitability was also determined.

### Mercury analysis at sub PPT levels by fluorescence: the next EPA frontier

*John Poling and Angelo Grillo, Questron Corporation, P.O. Box 2387 Princeton, NJ 08543, and Peter Stockwell, P.S. Analytical, B4 Chaucer Business Park, Watery Lane, Kemsing, Sevenoaks, Kent TN15 6QY, UK*

The present EPA Methods for Mercury require reporting of measurements between 0.2–2 ppb. As analytical chemists further explore the impact of mercury on the eco-system these levels have proven to be much too high. This has required a rethinking of what is an acceptable reporting level. Consideration and new method development has been undertaken to determine what can be done to analyse at lower levels with high precision and accuracy, without productivity suffering.

The application of fluorescence has provided such capabilities. Sensitivity down to 1–2 ppt, and less susceptibility to organic interferences which affect AA detection are two notable advantages of fluorescence technique.

This paper presented the application of a commercially available detector for mercury detection utilizing fluorescence. Detection and throughput of samples was discussed, as well as several applications now underway which will have significant impact on the levels of mercury detection.

EPA acceptance and timetables were reviewed, covering expected approval dates of low sensitivity methods and what impact these methods will have on detection requirements.

### Performance and applications of a new automated mercury analysis system

*Linda M. Zuvich and Timothy S. McDow, LDC Analytical, 3661 Interstate Park Road North, Riviera Beach, FL 33404*

Historically, automated analysis of mercury has been performed with cold vapor continuous-flow systems. A new mercury analysis system has been developed which automates the EPA cold vapor manual method. This system's unique design provides higher sensitivity, lower sample and reagent consumption, and superior performance and reliability over continuous-flow systems.

Current applications were presented to demonstrate the system's versatility. Data from a variety of samples including, soil, water and fish were used to show the system's flexibility in both range of sample matrix and concentration (low PPT to PPM).

Practical recommendations for obtaining the best possible sensitivity in mercury analysis was addressed, including optimal reagent selection for analysis and suggestions for maintaining sample stability at low concentrations of mercury.

The mercury analysis system's high sensitivity was highlighted with data showing instrument detection limits of 1 ppt. Data demonstrating the system's minimal carry-over level and superior precision, linearity and ease of use will also be presented.

### Mercury analysis from various samples using closed vessel microwave sample preparation

*Sara E. Littau, CEM Corporation, P.O. Box 200, 3100 Smith Farm Road, Matthews, NC 28106*

Mercury contamination in the environment has become an increasing concern due to the toxic nature of this element. The volatile nature of elemental mercury and organomercury compounds require the consideration of possible losses during sample preparation.

Digestion of environmental samples in closed vessels using microwave heating has become an accepted method of sample preparation prior to analysis for metals by AA or ICP.

This work described the digestion parameters required to prepare samples using closed vessel and microwave heating for the analysis of mercury using a cold vapour AA technique. The following standard reference materials and real world samples were digested and analysed: SRM 1575 Pine Needles, USEPA Metals in Fish, raw swordfish, municipal wastewater effluent, USEPA Dried Sludge, water motor oil and mixed solvent fuel. All of the real world samples were spiked with an organomercury standard.

Optimized considerations were presented for the sample size, acid and other reagents (concentration and volume), combined with the time, microwave power, temperature and pressure monitoring and control parameters needed to achieve quantitative digestions for analyses of mercury from these difficult samples. Particular attention was given to the cold vapor portion of the analysis. The effect that residual organic material has on the recoveries of mercury was discussed.

### Combining cold vapor generation with amalgamation to improve the detection limit of mercury in environmental samples

*Susan McIntosh and Joern Baasner, Perkin-Elmer Corporation, 761 Main Avenue, Norwalk, CT 06859-0219*

The determination of mercury in environmental samples has traditionally been performed using cold vapor atomic absorption. The detection limit of this technique is approximately 0.2 mg/l Hg. Recently the EPA approved a Flow Injection method, 245.1A, for the determination of mercury in water and waste water samples. This method provides increased sample throughput by automating the mercury vapour generation technique. The miniaturization of the manifold reduces reagent concentrations required, resulting in lower operating costs. The method detection limit, using an EDL System II, for drinking water and waste water samples in 0.06 mg/l Hg.

Mercury can be released into the environment from several sources, it is used as a preservative in paints, also in the manufacturing of battery cells and can be found in dental filling materials. Environmental requirements are necessitating the ability to determine mercury at ultra trace levels. The determination of mercury should ideally provide maximum sample throughput with the ultra trace detection.

A procedure for the determination of total mercury in environmental samples using a flow injection system coupled with an amalgamation accessory has been investigated. The use of amalgamation to preconcentrate mercury is based on the knowledge that mercury readily forms an amalgam with precious metals. The mercury vapour is carried by an argon gas stream from the gas liquid separator of the flow injection system to a quartz tube containing a gold/platinum gauze. The mercury collects on the gauze and, after a sufficient amount has been collected, the gauze is rapidly heated to drive off the mercury. The advantages of timed injection and volume injection are compared. The method detection limit of mercury in environmental samples, using the EPA approved method 245.1A, is significantly improved using this technique.

### EPA CLP automated mercury analysis of soils by cold vapor atomic absorption

*David N. Peterson, TMA/Skinner & Sherman Laboratories, Inc., 300 Second Avenue, Waltham, MA 02254*

Due to mercury's high level of toxicity and widespread distribution throughout the environment as a result of industrial and agricultural applications, sensitive automated mercury analysis remains an area of high interest in the environmental field. Mercury analysis has in the past been very labour intensive, and automation is not only a labour saving device, but by eliminating the human error associated with manual methods, it allows for an increased level of quality control.

The laboratory utilized the standard mercury digestion procedures as specified in EPA SW-846 Method 7471 or EPO 600 Method 245.5 along with an automated batch-processing system. The automated system consists of a mercury vapour generator, an auto-sampler, an elemental mercury detector, and an integrator. The mercury vapour generator adds the reducing agent to the reaction cell and bubbles carrier gas through the reducing agent in order to rid it of any mercury contamination while it fills the sample loop with the prepared sample from the autosampler. The sample is rinsed into the reaction cell with a 10% sulphuric acid solution. The system then bubbles carrier gas through the sample and the mercury vapour is carried through a drier tube into the Elemental Mercury Detector which measures the absorbance of the mercury vapour at 257.7 nm. The integrator records and integrates the peak and provides the results in concentration.

### The LIMS database: trends and future needs

*Robert Megargle, Cleveland State University, Cleveland, OH 44115*

A Laboratory Information Management System (LIMS) can be viewed as a database that is customized for use in the chemical testing laboratory. File structures are devised to hold typical data about customers, samples, test order, analytical results, quality control and management information. Input and output routines are added to the database to collect and present information in formats compatable to chemists. Instrument interfaces, bar code readers, and other schemes may be used to automate certain inputs.

Most laboratories view themselves as a place where samples are received, testing is done, and results are reported. The commercial LIMS of today is good for this kind of environment. Many laboratories, however, are beginning to understand that the real mission is more encompassing. The parent organization needs the analytical test results to make decisions, establish product quality, document adherence to regulations, or derive new knowledge. The purpose of the laboratory is not merely to turn our test results, it is to answer questions that are critically important to the business.

With this new perspective, the laboratory must re-orient itself to be a problem solver. The typical LIMS is less satisfactory in this context. Often unsatisfied is a need to save other information about a sample along with analytical test results. In a drug research program, for example, information about the subject animal, the drugs used, dosage history, and other such information is connected to each sample and is needed to understand the results. In environmental studies, the additional sample information might include site and weather conditions. The typical LIMS, however, has no database space allocated to save this additional data. There are no routines for putting it into the LIMS, printing it on reports, or using it to correlate test results. Another consequence of the global view is the increasing likelihood that the LIMS will be a part of a much larger information system.

It is unreasonable to expect a commercial LIMS supplier to meet all of these additional needs alone. Each site has a different set of problems that require customization for each business. One approach is participation by both the vendor and purchaser. The vendor provides tools that allow customized information systems to be built on top of a basic LIMS package that automates the more generic aspects of the laboratory. Purchasers accept responsibility for implementing and maintaining the customized part of the system. This approach has ramifications about the level of staff training required.

### LIMS—the next generation

*Jo S. Webber, Fisons Instruments, Danvers, MA 01923*

Laboratory Information Management Systems (LIMS) have been commercially available since the 1970s. Early LIMS applications acted simply as data repositories with little or no integration to other applications or instruments. Second generation LIMS appliations emerged in the late 1980s and early 1990s with the ability to handle much larger volumes of data, inter-system connectivity and instrument integration.

The evolution of LIMS into the 21st century has to address a number of driving forces including:

- The advancement of computer technology which has led to great improvements in networking capabilities. Close communication is now available between different information systems within an enterprise.
- The large amount of commercial hardware, databases, graphical user interfaces and operating systems currently available makes it difficult to predict the market front runners in 5, 10 or 20 years' time.
- Word processing and spreadsheet applications, in the past restricted to office use only, need to be used throughout an organization.
- Laboratories requirements are changing. Many laboratories are now undertaking a much wider range of work than in the past. Governing bodies and committees such as the EPA and FDA are imposing stricter quality control, documentation, validation and training requirements. There is a strong emphasis on the ownership and security of data.

This work addressed the requirements of the next generation of LIMS systems from both technological and functional aspects.

### The future of PC-based LIMS systems

*Cynthia de Sarno, Laboratory MicroSystems, Inc., 200 Broadway, Troy, NY 12180*

In the 1980s, the first LIMS were developed to meet regulatory require in the pharmaceutical industry. These early LIMS were based on mini-computers. With the advent of personal computers and local area network technology PC-based systems have become the fastest growing segment of the LIMS market.

PC-based systems are increasingly the platform of choice and are replacing mini-computer based systems for several reasons. First, capabilities which were available only on minicomputers are now available on PCs. Second, PC platforms are compatible with the cost effectiveness program that are driving many companies to right-size (down-size) information systems.

At the LIMS software level the trend is very clearly towards a graphically user interface. We have already seen systems introduced with a Microsoft Windows interface. X-Windows and Workplace Shell interfaces are not to far down the road.

PC-based LIMS software capabilities are increasing. Several software technologies are driving the increased capability. These include fault tolerant systems, dynamic data exchange (DDE), and binary large objects (BLOBs). These software technologies, among others, give LIMS increased ability to control the access to and disposition of all (including raw data), verbal data entry and feedback capability, chemical structure drawing, and natural language. Electronic laboratory notebooks and bi-directional communication with instruments, possibly based on the ADISS standard, are on the near horizon.

At the network operating system level, the future PC network operating systems would be derivatives of today's standards including Netware and UNIX, with increased capability in the areas, of portability, interoperability and multitasking. 32-bit versions of Windows and OS/2 have already been announced. PC-based client-server LIMS will also soon be available on both pure PC-based systems and mixed platform systems. In a mixed platform system a mini-computer is used as a server and PCs are used as front ends.

The development of standards outside of the laboratory is simultaneously changing the direction of PC based LIMS development and driving more companies and laboratories to implement PC-based LIMS. ISO9000, CLP and GALP will have increasing impact in the future.

The trend toward right sizing systems and the increased capability of PC-based platforms will lead to significant growth for the PC-based LIMS market in the future.

### Flow injection in the life sciences: a new approach to the study of living cells

*K. M. Scudder, G. D. Christian and J. Ruzicka, Department of Chemistry, University of Washington, Seattle, WA 98195*

Flow injection is widely used as a sample handling technique in analytical chemistry. The principles are applicable anywhere precise fluid handling is required. One such area is the study of adherent living cells by fluorescence microscopy. Significant advances have been made in microscopic techniques, the development of fluorescent probes for various cellular phenomena, and low-light-level imaging detectors for the weak fluorescence signals available. The manipulation of the fluid environment of the cells, however, has not had the same effort applied to it. Many studies are still done in open chambers on the microscope stage, with manual pipetting techniques used for reagent addition. Some microscope stage perfusion chamber designs exist, but their flow characteristics are usually secondary to other design constraints.

The authors applied sequential injection, a newer variant of the flow injection principle, to the perfusion of living cells on the microscope stage. In order to take advantage of the reproducibility of the sequential injection technique, a radial flow chamber called the fountain cell was developed. This chamber design, due to its flow symmetry, allows reproducible exchange of the liquid environment surrounding the cells while the cells are observed microscopically. Exchange time constants for the region of observation on the order of one second or better are easily obtained, with relatively low and controlled shear stress on the cells. Using the sequential injection technique, the cells can be exposed to a precisely timed sequence of reagents or stimulants, both in continuous flow and stopped flow modes. This combination of fluid handling precision and a chamber with known flow characteristics provides a new tool for studying rapid cellular responses to external chemical stimuli, such as the monitoring of cellular ion levels in response to surface receptor/ligand binding.

### How to squeeze high precision from segmented flow analysis. I. Instrumentation and software

*Donald A. Burns, Lawrence E. Wangen and Carolyn M. Huff, Los Alamos National Laboratory, Los Alamos, NM 87545*

For the best reproducibility in a continuous flow system, multiple measurements should be made at steady state. Hydraulic debubbling is typical of Segmented Flow Analysis (SFA), but by incorporating Bubble Thru The Flow Cell (BTTFC) technology, (a) steady state is reached sooner, (b) a cleaner system maintained, and (c) multiple measurements provided for. Peristaltic pump roller noise is cyclic and reproducible, so it can be virtually eliminated by averaging over an integer multiple of pump cycles. Other addressable sources of error include both long- and short-term drift, such as those caused by pump tubing and/or temperature changes.

Employing Hewlett Packard's HP-8452 diode array spectrophotometer, Alpkem's multichannel peristaltic pump, and fibre optics for remote operation, the authors have written software in QuickBASIC which can handle BTTFC in SFA. It performs these operations: (1) samples the signal every 100 msec, (2) produces a real-time display to monitor flowcell activity, (3) plots the analyte's entire spectrum each cycle, (4) accepts QC standards/samples as often as desired, (5) presents an on-screen scrolling list of sample concentrations in real time, and (6) prints a final report containing a variety of statistics. The tracing below is a 5-point standard curve followed by 10 replicates of the high standard (Nd as a stand-in for Pu). Current operating parameters for the analyser are as follows: cycle time 90 sec, bubble rate 90/min, sample/wash ratio 2/1, sample size 74 uL, and on-line dilution factor 21. Despite seemingly noisy peaks, precision was <0.05% RSD.

### Optimization of the analyses for volatile aromatics found in gasoline-contaminated samples using atomated static headspace

*Suya Wang, James D. Stuart, Department of Chemistry U-60, 215 Glenbrook Road, and Shi-Li Liu, Robert J. Carley, Environmental Research Institute U-210, University of Connecticut, Storrs, CT 06269-3060*

The Tekmar 7000/7050 Headspace Autosampler® was used to optimize the analyses of the volatile aromatic compounds in gasoline-contaminated groundwater and soil samples. Ten instrumental parameters were systematically varied using the Method Optimization Mode (MOM™) utility in conjunction with the 50 position carrousel autosampler. A megabore capillary column (0.53 mm i.d. 30 m in length and 3 micron film thickness od DB-1, J & W Scientific), was used in conjunction with a Hewlett-Packard 5890 Series II gas chromatograph equipped with a single flame ionization detector and a Hewlett-Packard 3396 Series II Integrator. Standard calibrations between 50 ppb and 20 ppm had linear correlation coefficients of at least 0.99 for the following compounds: benzene, toluene, ethylbenzene, *m*- and *p*-xylene (co-eluting), *o*-xylene, methyl-*t*-butyl ether, trichloroethane, trichloroethylene and tetrachloroethylene. Method detection limits for these compounds ranged from 7 to 40 ppb using the flame ionization detector. Good agreement was obtained for the analyses of the aromatic compounds when gasoline and spiked onto three, very different type soils and analysed using the Tekmar 7000/7050 Headspace Autosampler, a manual static headspace method and an EPA Method 8020 purge-and-trap/GC/MS method.

### Evaluation of an improved automated procedure for the measurement of weak acid dissociable and total cyanide

*Richard J. Berman, David Christmann, Curtiss Renn, Lea Rush and Bridget Scott, Perstorp Environmental Analysis, Inc., P.O. Box 648, Wilsonville, OR 97070*

The need to measure cyanide in many industrial and environmental applications is continuing to grow. This is due to its widespread presence in various fields such as the plating bath, steel, mining and petrochemical industries. The wastes from these processes are normally treated to destroy the most toxic cyanide species which are free cyanide and the weak acid dissociable cyanide complexes. Currently, most discharge permits require a report for only total cyanide. This is unfortunate since the total cyanide value does not give a realistic representation of the hazard of the waste by including other cyanide species which do not break down under normal environmental conditions. In addition, current standard procedures are complicated, time consuming, require the use of large amounts of hazardous reagents, and are subject to various interferences.

This paper described a simple automated analytical system to measure weak acid dissociable and total cyanide. Each sample is acidified on-line, irradiated with a UV lamp (total cyanide only), passed over a hydrophobic membrane for HCN diffusion into an alkaline receiving stream, and detected by the standard colorimetric procedure. The thiocyanate interference was eliminated by use of a new low wattage, long wavelength UV lamp. Samples from various industrial and environmental sources were analysed with the new system and the results compared to standard procedures EPA 335.3 and ASTM D4374. Amperometric detection for cyanide was also investigated as a way to eliminate the use of hazardous reagents.

In addition, new measurement alternatives for solid and difficult samples were presented.

### Automated colorimetric determination of chloride in water and wastewater by non-mercurimetric methods

*David R. Christmann, Richard Berman, Curtis N. Renn and Lea Rush, Perstorp Analytical Environmental, P.O. Box 648, Wilsonville, OR 97070*

In 1956, West and Coll originally described a procedure for the determination of chloride ion based on the formation of iron(III) chloro complexes in perchloric acid medium. Despite offering good sensitivity and relative freedom from interferences by other inorganic ions, this procedure has been largely ignored by the analytical chemistry community in favour of other colorimetric and titration techniques that typically make use of organic or inorganic mercury compounds. This was true until 1972 when, out of concern for laboratory safety and the mounting problem of managing and disposing of the hazardous laboratory waste, the ferric perchlorate procedure was modified for continuous flow analysis and applied to the determination of chloride in blood serum. Today the ferric perchlorate method has gained acceptance in clinical chemistry community and is approved by the FDA for clinical diagnostic measurement of chloride. However, it is still largely untested in applications in food, agriculture and environmental analysis.

In this paper, details of the ferric perchlorate procedure were reviewed and an automated method for the analysis of chloride in water and wastewater based on the procedure was described. Data were presented to illustrate the linearity, precision and accuracy of the method and its susceptibility to interferences. A direct comparison was made with the performance of the automated mercuric thiocyanate method, EPA 325.2.

### Automation equipment validation in the pharmaceutical industry

*Susan G. Kelley and Arthur L. Martin, Source For Automation, Inc., 115 Cedar Street, Milford, MA 01757*

With the purchase of a new piece of automated equipment in the pharmaceutical industry, QC laboratory managers must be concerned with validation. In selling automated equipment to and working with the major pharmaceutical companies throughout the world, the authors have found that there is some confusion as to what is required for validation, and that everyone approaches it a little differently. This paper was given to share information about validating automated equipment in the pharmaceutical industry.

The primary objectives of validation are to confirm that the piece of equipment was manufactured according to the PMA Life Cycle Development Plan, performs as it is intended, and successfully runs a particular product. The validation process is divided into four classifications: Manufacturing Qualification (MQ), Installation Qualification (IQ), Operation Qualification (OQ) and Product Qualification (PQ). The responsibility for performing these four functions is typically divided between the manufacturer and the pharmaceutical company. A document for each qualification type is usually required for full validation of the instrument.

### Determination of total arsenic in urine by flow injection-hydride generation atomic absorption spectrometry

*C. P. Hanna, J. F. Tyson, Chemistry Department, University of Massachusetts at Amherst, Amherst, MA 01003, and S. McIntosh, Inorganic Analysis Division, The Perkin-Elmer Corporation, 50 Danbury Road, Wilton, CT 06897*

For the determination of total toxic arsenic in urine, flow injection (FI)-hydride generation atomic absorption spectrometry (HGAAS) is a very appealing means of analysis. Beyond its ability to determine very low concentrations of arsenic in urine (less than 10 ppb), FI-HGAAS also features excellent sample throughput, greatly reduced sample size, less opportunity for sample contamination and enhanced tolerance for interfering elements when compared to that of batch systems. A potential problem in this determination is the different forms that arsenic can have as a result of transformation within the body. The arsenic can be in different inorganic oxidation states (+3 or +5), or in different stages of methylation (monomethylarsenic or dimethylarsenic). These differing arsenic species can subsequently be present in varying proportions within the urine sample. Since each arsenic species varies in sensitivity from direct conversion to their respective arsine, a digestion procedure is needed to convert all of the arsenic species to a single form. Furthermore non-toxic forms of arsenic, such as arsenobetaine and arsenocholine can present as a result of seafood consumption, can lead to overestimation of the toxic arsenic content of a urine sample.

A digestion procedure has been developed that quantitatively converts all of the toxic arsenic species noted above to As(V). Perchloric acid, which is frequently used for this determination, was not used due to the requirement of specialized exhausts and reported losses of arsenic. A rapid solid-phase extraction procedure removes arsenobetaine and arsenocholine prior to digestion, averting overestimation of the toxic arsenic portion. Off-line and on-line procedures for the pre-reduction of As(V) to As(III) were also developed. Quantitative recoveries of total arsenic in SRM 2670 (Toxic Metals in Urine) were obtained using both procedures, as well as spiked quantities of As(V) and dimethylarsenic in the presence of seafood-derived arsenic.

### Utilizing fluorescence for hydride elements with a commercial detector

*John Poling, Angelo Grillo, Questron Corporation, P.O. Box 2387, Princeton, NJ 08543, and Peter Stockwell, PS Analytical, B4 Chaucer Business Park, Watery Lane, Kemsing, Sevenoaks, Kent TN15 6QY, UK*

This paper discussed the use of fluorescence detection for hydride elements such as arsenic and selenium. The paper also presented a novel approach to developing a simple yet sensitive detector and continuous vapour generator for the automated detection of these elements. The Excalibur detector, developed by PS Analytical, incorporates continuous flame generation, a boosted discharge hollow cathode lamp, and a solar blind detector with fluorescence optics. A broad band interference filter can achieve sensitivity for detection of As at 0.5 ppb, Se at 0.1 ppb, Te at 2 ppb and Sb at 1.0 ppg. With specific filters detection limits show improvement by at least a factor of five.

Applications for the environment and industry were presented, along with limit of detection data for a variety of hydride elements.

### A new sample preparation method for the determination of sucralose in ketchup using dialysis, trace enrichment and high performance liquid chromatography

*N. M. Rafei, G. P. Cosgrove and C. M. Merkel, McNeil Specialty Products Company, 501 George Street, New Brunswick, NJ 08903-2400*

Previously, dialysis coupled with trace enrichment and high performance liquid chromatography has been used to assay biological materials in serum or plasma. In this work, the applicability of this sample preparation system to low-calorie ketchup was studied. A significant decrease (80%) in sample preparation time was achieved when compared to other standard extraction methods.

Samples were diluted (4:1) with deionized water and an internal of α-chloralose. Centrifugation at 9000 rpm for 20 minutes preceded injection of 500 μl of supernatant into the ASTED® (Automated Sequential Trace Enrichment of Dialysates) system. Dialyzation was performed using a 15 kD membrane. Elution from the trace enrichment cartridge (RP-18, 7 μ; 15 × 3.2 mm column) with 0.1 ml of 30 percent methanol (MeOH) and 70 percent water was followed by injection of 100 μl onto a $C_{18}$ 5 μ 10 cm analytical column. Analytes were eluted for 15 minutes with 30:70 MeOH:water at a rate of 1 ml/minute and detected using refractive index detection. Total time of sample preparation, detection and analysis was 50 minutes per sample.

Spike recoveries for the commercial concentration of sucralose in ketchup were performed in quadruplicate and averaged 99.7%, 104.4% and 99.8% respectively. Coefficients of variation were 1.6%, 3.9% and 0.4%. Further investigations have shown this method's applicability to different food matrices such as yogurt, cherry pie filling, relish and cordials.

### Fully automated sample analysis by matrix assisted laser desorption mass spectrometry

*J. P. Ricketts, M. P. Stevenson and J. S. Cottrell, Finnigan MAT Ltd, Hemel Hempstead, Hertfordshire HP2 4TG, UK*

Matrix assisted laser desorption mass spectrometry has become a widely accepted technique for the analysis of peptides, proteins, carbohydrates and related materials. Time per analysis can be as short as three or four minutes, most of which is the time taken to achieve a working vacuum after the sample has been introduced into the instrument.

This process can be accelerated by loading multiple samples onto a single substrate so that they can be introduced into the vacuum system simultaneously. While this is a valuable improvement, it falls short of true automation in at least two respects. Firstly, it does not address the time consuming process of depositing the sample and matrix solutions onto the substrate. Secondly, it is essential to optimize the laser power for each sample individually if good quality spectra are to be obtained.

The authors have developed an automatic sample handling system for the Finnigan Lasermat which remedies these shortcomings. It has been designed to be fitted to a standard instrument in place of the single sample introduction mechanism, and accepts a substrate carrying up to 48 samples.

An automatic analysis may be interrupted for the introduction of a single urgent sample and then resumed. The software uses a spreadsheet metaphor to enable all instrument control and data processing parameters to be manipulated for individual samples while still providing an overview of the entire analysis at a glance. Throughput has been benchmarked at just under one sample per minute.

The feasibility of coupling this device to an HPLC or CZE, so as to eliminate the manual collection and preparation of large numbers of fractions, was discussed.

### Adapting the control of microwave digestion for highly organic samples

*Mike Moses, Questron Corporation, P.O. Box 2387, Princeton, NJ 08543*

Modern microwave digestion instrumentation has proven itself to be an invaluable tool for rapid sample preparation. Instantaneous heating provided by microwave energy coupled with pressurized digestion vessels allow digestions to be carried out in minutes, rather than hours.

The limitations of these systems has been their usefulness for highly organic samples. Under conditions of elevated temperatures and pressures, the reaction between sample and acid can quickly go out of control. Because of these highly exothermic reactions, and older microwaves' inability to adapt power to rapidly changing conditions, sample size for some organic samples had to be severely restricted. In fact, some highly organic sample types were considered undoable by pressurized microwave systems.

New microwave digestion systems, which are now commercially available, offer adaptive control of digestion rates for highly reactive samples. This paper explored the use of algorithmically controlled microwave heating in conjunction with a variety of new chemistries to achieve the total digestion of difficult sample types.

### Automated continuous flow microwave dissolution: rapid unattended sample preparation

*Edward E. King, Douglas Ferguson, Brian Renoe and David Barclay, CEM Corporation, 3100 Smith Farm Road, P.O. Box 200, Matthews, NC 28106-0200*

Changes in chemical instrumentation have improved analytical capabilities for many processes in the laboratory. Automated features of many instruments allow unattended use for routine analyses, but sample preparative procedures have not seen improvements of this type. Further, while the introduction of microwave heating for sample decomposition dramatically decreased sample dissolution time, it did not eliminate some of the time consuming and labour intensive tasks. To address these omissions, the authors developed dynamic flow through microwave instrumentation and methodology which allows automation to be extended to sample decomposition procedures. Samples are simply placed on the autosampler and later removed as completely digested samples—ready for analysis.

This paper outlined the development of the methodology and instrumentation from the research prototype to a fully automated system. The novel approaches implemented in the instrumentation were explained to ensure satisfactory progress of the samples through the system. The use of non-invasive sensor mechanisms on the system allows samples to be monitored through the instrument and dispensed ready for analysis completely without contamination.

The results of an extensive study of the digestion conditions achievable with a variety of acids and acid concentrations were presented. This work includes data for pressure and temperature taken along the path of the flowing sample as it is processed from sample injection through the digestion to subsequent collection. These data demonstrate the consistent performance of the system for all sample types. This performance is compared to closed vessel microwave sample dissolution and demonstrates the superiority of the flow system. The description of the process and the results also demonstrate the inherent safety of the technique for handling dissolution reagents. Finally, the data show a minimization of contamination for the flow technique compared to conventional methodologies.

A discussion of the results showed the precision and accuracy of the technique for a range of elemental determinations on selected samples.

Finally, the application of the flow sample preparation technique to other sample type was demonstrated with metal analyses data. The focus was on sample matrices that are traditionally difficult to prepare. These matrices include samples with largely organic components such as oil, fatty tissue, and mixed waste samples.

### A new automated total organic halide (tox) analyser

*William L. Robinson, Gwen Ohlson and Yoshi Takahashi, Rosemount Analytical Inc., Dohrmann Division, 3240 Soctt Boulevard, Santa Clara, CA 95054*

The environmental group parameter Total Organic Halide (TOX), pioneered by the Dohrmann Division of Rosemount Analytical Inc., has become a widely accepted indicator of the total organic pollution in drinking, ground, well, and waste waters. This method consists of several steps. TOX employs absorption of the organic halid onto granular activated carbon (GAC) followed by combustion of the GAC and microcoulometric detection of the halide. This method is effective, but can be labour intensive.

This paper described an automated halide analyser which employs an improved combustion/detection system designed to meet the broad scope of current applications and reduce maintenance. The analyser also features a new friendly user interface and an enhanced data handling system. An optional autosampler has been designed to free the operator from having to manually inject in the GAC columns into the combustion system.

Data were presented to illustrate the wide dynamic range and performance of this new instrument for several environmental applications.

